# Multivariate Bayesian Dynamic Borrowing for Repeated Measures Data With Application to External Control Arms in Open‐Label Extension Studies

**DOI:** 10.1002/bimj.70079

**Published:** 2025-10-07

**Authors:** Benjamin F. Hartley, Matthew A. Psioda, Adrian P. Mander

**Affiliations:** ^1^ Veramed Ltd. Twickenham UK; ^2^ Department of Biostatistics GSK Research and Development Collegeville Pennsylvania USA; ^3^ Department of Biostatistics GSK Research and Development London UK

**Keywords:** clinical trial design, external control arm, historical data, informative prior, multivariate analyses, robust mixture priors

## Abstract

Borrowing analyses are increasingly important in clinical trials. We develop a method for using robust mixture priors in multivariate dynamic borrowing. The method was motivated by a desire to produce causally valid, long‐term treatment effect estimates of a continuous endpoint from a single active‐arm open‐label extension study following a randomized clinical trial by dynamically incorporating prior beliefs from a long‐term external control arm. The proposed method is a generally applicable Bayesian dynamic borrowing analysis for estimates of multivariate summary metrics based on a multivariate normal likelihood function for various parameter models, some of which we describe. There are important connections to estimation incorporating a prior belief for a hypothetical estimand strategy, that is, had the event not occurred, for intercurrent events which lead to missing data.

## 1 Introduction

The term Bayesian dynamic borrowing is used to describe a family of methods which allow for the incorporation of historical data, that is, prior information, into an analysis of current data. The amount of information contributed by the prior depends on the degree of commensurability between the current and historical data (Viele et al. 2014). The motivating purpose of Bayesian dynamic borrowing, as it pertains to clinical drug development, is that there may be uncertainty about the relevance of historical data to the current study data due to a drift in clinical outcomes, for example, because of improved standard‐of‐care or changes in the affected population. Incorporating historical data via dynamic borrowing from external control arms is increasingly common to support submissions for drug approvals (Goring et al. 2019). This is particularly important in orphan or pediatric indications where recruitment to a randomized trial may be difficult, and comparisons would otherwise be underpowered (FDA 2020). However, there is an additional opportunity for the more general application of dynamic borrowing in estimating treatment effects from long‐term, single‐arm open‐label extensions to randomized trials where the validity of treatment effect estimates has been questioned (Taylor and Wainwright 2005; Megan et al. 2012).

In open‐label extension studies, efficacy outcomes are usually collected and descriptively summarized, but formal analyses of long‐term treatment effectiveness are difficult because long‐term randomization to a control may not be ethical and so in most open‐label extension studies control‐arm participants from the randomized trial receive the active treatment. Wang et al. (2022) searched clinicaltrials.gov for open‐label extension studies from 2009 to 2019 and identified 1115 studies, of which only seven (0.6%) used an external control arm. The majority (62.2%) originated from randomized controlled trials and were principally designed to support drug development through the collection of additional long‐term safety data. The authors conclude that

The contrast of the large and growing number of uncontrolled extensions with the small number of studies that utilized [external controls] showed clear opportunities for enhancement in design, measurement, and analysis of uncontrolled extensions to allow causal inferences on long‐term treatment effects.

Using control data from the randomized trial and dynamically borrowing information from a set of external controls may therefore be of great value in improving the validity of treatment effect estimates from open‐label extension studies.

Various methods exist to dynamically incorporate historical data as part of a prior, including power priors (Chen and Ibrahim 2000), power priors modified through normalization to avoid violation of the likelihood principle (Duan et al. 2006; Neuenschwander et al. 2009), commensurate priors (Hobbs et al. 2011), and robustified meta‐analytic predictive (MAP) priors (Schmidli et al. 2014). Power priors and modified versions thereof share a similar structure in which the historical data likelihood function is weighted by an exponent that can be specified, estimated (Bennett et al. 2021), or given a prior distribution. MAP priors (Spiegelhalter et al. 2004; Neuenschwander et al. 2010) are informative priors derived from historical data based on a meta‐analysis using a hierarchical model to account for between‐trial heterogeneity. Schmidli et al. (2014) proposed a modification to MAP priors to dynamically borrow historical data. They suggest approximating the MAP prior with a mixture of conjugate prior distributions, and then, in order to robustify the prior (Lindley 1968; Dawid 1973; O'Hagan 1979), they add a heavy‐tailed vague component to the mixture prior. For the remainder of this paper, we refer to the robustified MAP prior as a robust mixture prior and assume, without loss of generality, that the informative component is a single distribution. This method for dynamically borrowing historical data is attractive because of the ease of characterization of the prior as a mixture of well‐known distributions and because analysis with a mixture of conjugate priors emits a closed‐form posterior distribution.

In this paper, we introduce Bayesian dynamic borrowing with a robust mixture prior for *multivariate* data and show how borrowing historical data from an external control arm, collected over an extended follow‐up period, can be used to provide a valid comparator to evaluate efficacy from a shorter term randomized trial with an open‐label extension phase. We demonstrate borrowing using both a continuous‐time, that is, intercept‐slope analysis model, and a categorical‐time, that is, by‐visit analysis model. For the latter, the data collection visits of the current study (randomized trial and open‐label extension) and historical study (external control) must occur at similar times; otherwise, the visits need only span a common time period. The motivation for our work is to illustrate how a causally valid estimate of a long‐term treatment effect can be obtained from a single‐arm open‐label extension when the external controls and current study participants are exchangeable.

The motivating example is described in detail in Section 2. In Section 3, the methods used to extend robust mixture priors to multivariate normal data are presented with some theoretical results for the calculation of closed‐form posterior distributions. The methods are illustrated with an example and a simulation study in Section 4. The example compares three different mixture priors. In the simulation study, the motivating example study design from Section 2 is used to assess intercept‐slope and by‐visit analysis models for different levels of prior‐current data conflict. Finally, there is a discussion of some of the practical considerations when applying the method in Section 5.

## 2 Motivating Study Design

The motivating study is a planned 52‐week randomized double‐blind trial comparing active treatment, n=150, with a placebo control, n=150, in participants with a rare chronic disease. In such a rare and life‐threatening disease, it may be unethical to continue treatment with a placebo after 52 weeks, and so after completing the randomized controlled trial, participants will be offered entry into an open‐label extension, during which all participants will be given the active treatment. Participants will be followed up for at least an additional 52 weeks. The primary endpoint is a continuous lung function measure, forced vital capacity (FVC). Dynamically borrowing multivariate data from an external control arm using a robust mixture prior will enable a treatment comparison of FVC between active and control arms at 104 weeks despite there being only 52 weeks of control data in the randomized trial and its open‐label extension.

**FIGURE 1:**
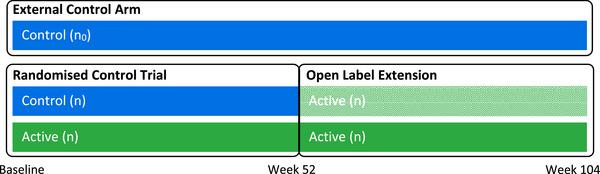
Study design of motivating example.

A schematic of the planned design is shown in Figure 1. The *current data* will come from a two‐arm randomized study of an active treatment and a control with n participants per arm, followed by an open‐label extension during which all participants will receive the active treatment. The open‐label extension data from participants who were randomized to control (light green data in the figure) will not contribute to this proposed borrowing analysis. The *historical data* will be an external control arm with $n_{0}$ participants. Although referred to as historical data, the external control arm might be retrospective or contemporaneous with the randomized trial, that is, followed up prospectively. The posteriors for the relevant mean parameters from the external control arm may be standardized using a variety of methods to better reflect the current study population using baseline characteristics (Lin et al. 2019), but this is not the focus of our work.

If the current control data in the randomized trial are consistent with the prior predictive distribution of the informative component derived from historical control data over the period of follow‐up for the randomized trial, then the posterior distribution for the control arm should reflect substantial borrowing from the historical data, enabling a more precise estimate of the treatment effect. If the current control data in the randomized trial are not well predicted by the informative component, then the posterior distribution for the control group will be more consistent with that obtained from an analysis with the vague component of the prior and the credible interval for the treatment comparison will be correspondingly less precise.

The proposed method has important connections to the hypothetical estimands strategy discussed in the E9 R1 addendum (ICH 2020) for the “by‐design intercurrent event” of treatment‐switching at the end of the randomized trial, that is, quantifying the treatment effect at the 104‐week visit had treatment‐switching not taken place. The treatment policy estimand with regard to treatment switching after 52 weeks is of less interest since the offer to move onto active treatment applies only to the control arm. Additionally, by constructing a treatment effect estimate with historical borrowing based on commensurability with the randomized trial data, the population for which the estimand is defined can be the randomized trial entry population and not the self‐selecting open‐label extension entry population.

## 3 Methods

### 3.1 Notation

The data for i=1,2,…,n participants in the current study randomized control arm are denoted by $x_{i}$. The $x_{i}$ are assumed to follow a normal distribution with mean μ and positive‐definite covariance matrix Σ. The dimension of the $x_{i}$ is denoted J. For a by‐visit model, the mean is allowed to differ at each of the J time points with no additional structure imposed. This type of mean structure is commonly used in fitting mixed models for repeated measures. For an intercept‐slope model, the mean outcome is assumed to satisfy a linear trajectory over time characterized by the J=2 intercept and slope parameters. The inverse of the covariance matrix $\Sigma^{-1}$ is called the precision matrix. Denote the multivariate normal density function by φ, parameterized by a mean vector and a covariance matrix. The $x_{i}$ are independent and identically distributed according to $\varphi \left(x_{i};\mu ,\Sigma \right)={\left(2\pi \right)}^{-\frac{J}{2}}{\left|\Sigma \right|}^{-\frac{1}{2}}\exp\left(-\frac{1}{2}{\left(x_{i}-\mu \right)}^{T}\Sigma^{-1}\left(x_{i}-\mu \right)\right)$. The multivariate normal likelihood function for outcome data given a mean and covariance matrix is defined as follows:

(1) $$\begin{array}{c} \prod_{i=1}^{n}\varphi \left(x_{i};\mu ,\Sigma \right)=\prod_{i=1}^{n}{\left(2\pi \right)}^{-\frac{J}{2}}{\left|\Sigma \right|}^{-\frac{1}{2}}\exp\left(-\frac{1}{2}{\left(x_{i}-\mu \right)}^{T}\Sigma^{-1}\left(x_{i}-\mu \right)\right). \end{array}$$

Prior belief about the distribution of the mean μ can similarly be expressed using the multivariate normal density function with two hyperparameters, a mean vector $\mu_{0}$ and a covariance matrix $\Sigma_{0}$ as follows,

(2) $$\begin{array}{c} \varphi \left(\mu ;\mu_{0},\Sigma_{0}\right)={\left(2\pi \right)}^{-\frac{J}{2}}{\left|\Sigma_{0}\right|}^{-\frac{1}{2}}\exp\left(-\frac{1}{2}{\left(\mu -\mu_{0}\right)}^{T}{\Sigma_{0}}^{-1}\left(\mu -\mu_{0}\right)\right). \end{array}$$

Prior belief about a covariance matrix Σ can be specified using the inverse‐Wishart distribution density function ω, with two hyperparameters, a scale matrix $\Psi_{0}$, and degrees of freedom $\nu_{0}$,

(3) $$\omega \left(\Sigma ;\Psi_{0},\nu_{0}\right)=\frac{{\left|\Psi_{0}\right|}^{\frac{\nu_{0}}{2}}{\left|\Sigma \right|}^{-\frac{\nu_{0}+J+1}{2}}}{2^{\frac{\nu_{0}J}{2}}\Gamma_{J}\left(\frac{\nu_{0}}{2}\right)}\exp\left(-\frac{1}{2}\mathrm{tr}\left(\Psi_{0}\Sigma^{-1}\right)\right),$$

where $\Gamma_{J}$ is the multivariate gamma function. This parameterization, from Gelman et al. (1995), implies that if Σ is inverse‐Wishart distributed with a scale matrix $\Psi_{0}$ and $\nu_{0}$ degrees of freedom, then $\Sigma^{-1}$ is Wishart distributed with scale matrix ${\Psi_{0}}^{-1}$ and $\nu_{0}$ degrees of freedom. With these definitions, a joint prior for (μ,Σ) can be specified by multiplying the density functions in (2) and (3) to give a product prior density function $\varphi \left(\mu ;\mu_{0},\Sigma_{0}\right)\omega \left(\Sigma ;\Psi_{0},\nu_{0}\right)$. The distributions are, by construction, separable, and marginally the mean vector is normally distributed $\varphi (\mu ;\mu_{0},\Sigma_{0})$ and the covariance matrix is inverse‐Wishart distributed $\omega (\Sigma ;\Psi_{0},\nu_{0})$. Alternatively, the multivariate normal prior for the mean vector μ can be specified to be conditional on the covariance matrix Σ to create a normal‐inverse‐Wishart joint prior distribution which has four hyperparameters, a mean vector $\mu_{0}$, a hyperparameter which scales the covariance matrix $\lambda_{0}$, a scale matrix $\Psi_{0}$, and degrees of freedom $\nu_{0}$ such that the joint density function is $\varphi \left(\mu ;\mu_{0},\Sigma /\lambda_{0}\right)\omega \left(\Sigma ;\Psi_{0},\nu_{0}\right)$. In this case, the marginal distribution of the covariance matrix is $\omega (\Sigma ;\Psi_{0},\nu_{0})$, but the marginal distribution of the mean vector is a multivariate t‐distribution with mean $\mu_{0}$, scale matrix $\Psi_{0}/\left[\lambda_{0}\left(\nu_{0}-J+1\right)\right]$, and $\nu_{0}-J+1$ degrees of freedom (Gelman et al. 1995).

Denote p(μ,Σ|x) as the posterior distribution that is, the probability distribution of the parameters (μ,Σ) given all the observed data x, then p(μ,Σ|x)=p(μ,Σ)p(x|μ,Σ)/p(x), where p(μ,Σ) is the prior distribution of the parameters, p(x|μ,Σ) is the likelihood function of the data, and p(x) is a normalizing constant called the marginal likelihood or model evidence. If the covariance matrix Σ is known (or is assumed to be equal to a plug‐in estimator $\hat{\Sigma }$), and a prior belief about μ is specified using the multivariate normal distribution (2) and updated based on the likelihood function from (1), then the posterior density function for μ is also multivariate normal and is given by $p\left(\mu |x\right)=\varphi \left(\mu ;{\mu_{0}}^{\prime },{\Sigma_{0}}^{\prime }\right)$, where ${\mu_{0}}^{\prime }={\left({\Sigma_{0}}^{-1}+n\Sigma^{-1}\right)}^{-1}\left({\Sigma_{0}}^{-1}\mu_{0}+n\Sigma^{-1}\bar{x}\right)$ and ${\Sigma_{0}}^{\prime }={\left({\Sigma_{0}}^{-1}+n\Sigma^{-1}\right)}^{-1}$ (Gelman et al. 1995). Similarly, when a joint prior belief about (μ,Σ) is specified using the normal‐inverse‐Wishart distribution and updated based on the likelihood function from (1), then the posterior density function for (μ,Σ) is also normal‐inverse‐Wishart and is given by $p\left(\mu ,\Sigma |x\right)=\varphi \left(\mu ;{\mu_{0}}^{\prime },\Sigma /\lambda_{0}^{\prime }\right)\omega \left(\Sigma ;{\Psi_{0}}^{\prime },\nu_{0}^{\prime }\right)$, where ${\mu_{0}}^{\prime }=\left(\lambda_{0}\mu_{0}+n\bar{x}\right)/\left(\lambda_{0}+n\right)$, ${\Psi_{0}}^{\prime }=\Psi_{0}+\left[\sum_{1}^{n}\left(x_{i}-\bar{x}\right){\left(x_{i}-\bar{x}\right)}^{T}\right]+\left(\lambda_{0}n/\left(\lambda_{0}+n\right)\right)\left(\mu_{0}-\bar{x}\right){\left(\mu_{0}-\bar{x}\right)}^{T}$, $\lambda_{0}^{\prime }=\lambda_{0}+n$, and $\nu_{0}^{\prime }=\nu_{0}+n$ (Gelman et al. 1995).

### 3.2 Multivariate Robust Mixture Priors

A mixture prior density is a convex combination of density functions, that is, a weighted sum of density functions in which the weights sum to 1. Let the weights be indexed by c, then $\sum_{c}w_{c}=1,w_{c}>0\;\forall \;c$. In what follows, we use priors with two components c∈{inf,vag}, an informative component, and a vague component. This describes a situation in which there is evidence from a historical control arm but uncertainty regarding the degree of applicability of that information to the current study. From a purely subjectivist Bayesian perspective, the weight $w_{\text{inf}}$ assigned to the informative component and hence its complement $w_{\text{vag}}=1-w_{\text{inf}}$ assigned to the vague component represent one's a priori belief about which component distribution best describes the parameters in the model. However, in practice, weights are often chosen so that analyses have favorable frequentist properties, for example, ensuring that the (average) probability of a declaration of efficacy is not unreasonably inflated when the null hypothesis holds (Best et al. 2023).

Mixture priors constructed with a vague component to robustify analyses to prior‐current data conflict have been described by Schmidli et al. (2014) for a range of univariate family distributions. Here, we construct mixtures of *multivariate* prior distributions, optionally involving nuisance parameters. We propose three different possible constructions for the prior.

i. The covariance matrix Σ is known, and the prior on the mean μ is a mixture of two multivariate normal distributions,
(4) $$\begin{array}{c} p\left(\mu \right)=w_{\text{inf}}\varphi \left(\mu ;\mu_{\inf},\Sigma_{\inf}\right)+w_{\text{vag}}\varphi \left(\mu ;\mu_{\mathrm{vag}},\Sigma_{\mathrm{vag}}\right). \end{array}$$
ii. The covariance matrix Σ is unknown, and the joint prior on the mean and covariance parameters (μ,Σ) is specified as a mixture of two normal‐inverse‐Wishart distributions,
(5) $$\begin{array}{cc} p(\mu ,\Sigma )=\; & w_{\text{inf}}\varphi \left(\mu ;\mu_{\inf},\frac{\Sigma }{\lambda_{\text{inf}}}\right)\omega \left(\Sigma ;\Psi_{\inf},\nu_{\text{inf}}\right)\\ & +w_{\text{vag}}\varphi \left(\mu ;\mu_{\mathrm{vag}},\frac{\Sigma }{\lambda_{\text{vag}}}\right)\omega \left(\Sigma ;\Psi_{\mathrm{vag}},\nu_{\text{vag}}\right). \end{array}$$
iii. The covariance matrix Σ is unknown, and the joint prior on the mean and covariance parameters (μ,Σ) is specified as the mixture of two products of independent multivariate normal and inverse Wishart distributions,
(6) $$\begin{array}{cc} p(\mu ,\Sigma )=\; & w_{\text{inf}}\varphi \left(\mu ;\mu_{\inf},\Sigma_{\inf}\right)\omega \left(\Sigma ;\Psi_{\inf},\nu_{\text{inf}}\right)\\ & +w_{\text{vag}}\varphi \left(\mu ;\mu_{\mathrm{vag}},\Sigma_{\mathrm{vag}}\right)\omega \left(\Sigma ;\Psi_{\mathrm{vag}},\nu_{\text{vag}}\right). \end{array}$$

The assumption that the covariance matrix of the data is known, made for prior (i) is commonly used to facilitate study design simulations of large sample sizes where the sample covariance matrix will be a good estimate of the true covariance matrix. If the sample size of the current data is not sufficient to treat the covariance as known for computational expediency, the covariance can be modeled and a prior assigned to it, for example, using priors (ii) or (iii). Priors (i) and (ii) exploit the conjugacy between the multivariate normal data model and prior distributions and result in closed‐form posteriors. The posterior resulting from analysis with prior (iii) does not have a closed form but can be characterized efficiently with Markov chain Monte Carlo (MCMC) sampling. Indeed, if the conjugacy requirement is dropped, there is no obligation to use multivariate normal or inverse‐Wishart priors on the mean and covariance, respectively, but these distributions are still commonly used.

The posterior distribution for μ and Σ from the analysis of a historical control arm of $n_{0}$ controls can be taken as the informative component in the prior mixture distribution. The vague component can be constructed by downweighting the informative component such that the vague component represents the evidence from a single representative participant from the historical control data (Callegaro et al. 2023). For priors (i) and (iii), it is therefore convenient to choose the mean vector and covariance matrix hyperparameters for the vague component on the mean parameters as $\mu_{\mathrm{vag}}=\mu_{\inf}$ and $\Sigma_{\mathrm{vag}}=n_{0}\Sigma_{\inf}$. Similarly, for prior (ii), define hyperparameters $\mu_{\mathrm{vag}}=\mu_{\inf}$ and $\lambda_{\text{vag}}=\lambda_{\text{inf}}/n_{0}$ so that for all the priors considered, the information contained in the vague component on the mean is that of a single representative participant. It is not a requirement that the means of the informative and vague components $\mu_{\inf}$ and $\mu_{\mathrm{vag}}$, be equal, nor that the covariance matrix of the vague component $\Sigma_{\mathrm{vag}}$ be a multiple of the covariance matrix of the informative component $\Sigma_{\inf}$. However, the robustness of the posterior to a prior‐current data conflict depends on the heavy tails of the vague component distribution (Schmidli et al. 2014). The vague component can be made arbitrarily flat, for example, by multiplying the covariance matrix of the informative component by a large enough number, but it cannot be improper. It may not be intuitive to use the information from the historical controls on the mean outcome μ in the informative component while disregarding the historical information on the covariance matrix Σ, but, in the applications shown, we choose to do so for the following reasons. First, the principle interest in these models is to make inferences on the mean parameters, and it does not seem desirable to downweight the informative component due only to a drift in the covariance parameters between the historical controls and the current controls. (The concept of “partial borrowing” has been considered before by Psioda and Ibrahim 2019.) Second, the impact on the marginal prior induced on the mean is not substantial (i.e., moving from a t‐distribution to a normal distribution) unless the sample size of the current controls is very small. Therefore, using fairly diffuse inverse‐Wishart distributions for the informative and vague components in priors (ii) and (iii) seems reasonable.

Bayesian analysis with a mixture prior results in a mixture posterior (Bernardo and Smith 2000). The posterior distributions associated with prior (i) (Equation 4), prior (ii) (Equation 5), and prior (iii) (Equation 6) are, therefore, all mixture distributions described by a convex combination of updated density functions and updated weights. In the posterior, these weights are updated using the current control data, whereafter they characterize the posterior probability that the current control data arose from the prior predictive processes defined by the informative and the vague prior components. The updated component density functions of the posterior are described in Section 3.3, and the updated weights are described in Section 3.4.

### 3.3 Posterior Distribution Components

When the prior and the analysis model share a common dimension J, either because the models are both intercept–slope models with just two parameters, or in the context of by‐visit models, because the historical and current control arm data are collected at the same set of time points, then the posterior distribution components are well known. For priors (i) and (ii), the posterior distribution components are multivariate normal and normal‐inverse‐Wishart, respectively, with the updated hyperparameters that have previously been described in Section 3.1. However, a prior may be defined on a higher dimension, J+K, than the dimension of the analysis model. In this case, the density functions given in (2) and (3) will have J replaced by J+K. The posterior components arising from a J+K dimension prior from historical data and a J dimension likelihood function on current data using priors (i) and (ii) are given in this section. Proofs, using Bayes' theorem, can be found in the Supporting Information.

For prior (i), the posterior can in fact, be constructed through augmentation of current data from J to J+K dimensions, the procedure is described in Box 1. This enables the posterior components of dimension J+K to be simply written down using the conjugate result in Section 3.1.

**Box. 1: Constructing the Posterior From Lower Dimension Data and a Multivariate Normal Prior on the Mean Parameter**

From the observed data in the current study, estimate the mean vector $\bar{x}$ of length J, and a J×J covariance matrix $\hat{\Sigma }$. Invert this matrix to create an estimated precision matrix and extend the precision matrix with zeros to be a J+K by J+K matrix. This precision matrix then additionally describes the precision of data at the unobserved K visits, since data at these visits are indeed unobserved. The off‐diagonal zeros in the augmented precision matrix can be heuristically justified because the unobserved data are conditionally independent of the observed data (if it does not exist, it adds no additional information). Denote this zero‐augmented precision matrix ${\tilde{\Sigma }}^{-1}$. The mean vector $\bar{x}$ of the current control arm also needs to be extended to the same J+K dimensions as the prior, $\varphi (\mu ;\mu_{0},\Sigma_{0})$. Call this extended vector $\tilde{x}$. The K values added can be arbitrary because, in the posterior density function, they are always multiplied by zeros from ${\tilde{\Sigma }}^{-1}$. The zero‐augmented precision matrix and extended mean vector can be written as follows:

$$\begin{array}{cc} {\tilde{\Sigma }}^{-1}=\left(\begin{array}{cc} {\hat{\Sigma }}^{-1} & 0_{J\times K}\\ 0_{K\times J} & 0_{K\times K} \end{array}\right), & \tilde{x}=\left(\begin{array}{c} \bar{x}\\ \vdots \end{array}\right). \end{array}$$

The posterior can be written as follows using the known conjugate result since the prior and likelihood function now both have dimension J+K,

(7) $$\begin{array}{cc} p(\mu |x) & =\varphi \left(\mu ;{\left({\Sigma_{0}}^{-1}+n{\tilde{\Sigma }}^{-1}\right)}^{-1}\left(\mu_{0}{\Sigma_{0}}^{-1}+n{\tilde{\Sigma }}^{-1}\tilde{x}\right),{\left({\Sigma_{0}}^{-1}+n{\tilde{\Sigma }}^{-1}\right)}^{-1}\right). \end{array}$$

For practical purposes, (7) is sufficient to write down the posterior because the values added to create the full $\tilde{x}$ vector are irrelevant. However, more formally, to avoid notation involving a partially defined observed mean vector, and to understand the posterior as the solution to a missing data problem, partition the mean vector μ, and its prior $\varphi (\mu ;\mu_{0},\Sigma_{0})$ into blocks corresponding to observed and unobserved data such that $p\left(\mu \right)=\varphi \left(\mu ;\mu_{0},\Sigma_{0}\right)=\varphi \left((\begin{array}{c} \alpha \\ \beta \end{array});(\begin{array}{c} \alpha_{0}\\ \beta_{0} \end{array}),(\begin{array}{cc} A_{0} & B_{0}\\ C_{0} & D_{0} \end{array})\right)$. The unknown parameter vectors α and β are means of the current control arm data at the observed and unobserved visits, respectively. The prior value vectors and matrices $\alpha_{0}$, $\beta_{0}$, $A_{0}$, $B_{0}$, $C_{0}$, and $D_{0}$ are the conformable blocks of $\mu_{0}$ and $\Sigma_{0}$, respectively. Note that $B_{0}={C_{0}}^{T}$, and both $A_{0}$ and $D_{0}$ are symmetric. Define also $S_{0}=D_{0}-C_{0}{A_{0}}^{-1}B_{0}$ the Schur complement of $A_{0}$ in $\Sigma_{0}$. The posterior distribution for μ given in (7) can then be written as follows:

(8) $$\begin{array}{ccc} p\left(\mu |x\right) & = & \varphi \left(\left(\begin{array}{c} \alpha \\ \beta \end{array}\right);\left(\begin{array}{c} {\alpha_{0}}^{\prime }\\ \beta_{0}+C_{0}{A_{0}}^{-1}\left({\alpha_{0}}^{\prime }-\alpha_{0}\right) \end{array}\right),\right.\\ & & \left.\left(\begin{array}{cc} {A_{0}}^{\prime } & {A_{0}}^{\prime }{\left(C_{0}{A_{0}}^{-1}\right)}^{T}\\ C_{0}{A_{0}}^{-1}{A_{0}}^{\prime } & S_{0}+C_{0}{A_{0}}^{-1}{A_{0}}^{\prime }{\left(C_{0}{A_{0}}^{-1}\right)}^{T} \end{array}\right)\right), \end{array}$$

where primes denote the usual posterior hyperparameters ${\alpha_{0}}^{\prime }={\left({A_{0}}^{-1}+nA^{-1}\right)}^{-1}\left({A_{0}}^{-1}\alpha_{0}+nA^{-1}\bar{x}\right)$ and ${A_{0}}^{\prime }={\left({A_{0}}^{-1}+nA^{-1}\right)}^{-1}$. The marginal distribution on the first J parameters p(α|x) is the familiar $\varphi (\alpha ;{\alpha_{0}}^{\prime },{A_{0}}^{\prime })$. The marginal distribution of the last K parameters p(β|x) is the distribution of the mean at visits where the data is, by construction, all missing. The posterior means of the last K parameters have been shifted from the prior means $\beta_{0}$ by an amount which depends on the difference between the posterior and prior mean parameters for the observed data ${\alpha_{0}}^{\prime }-\alpha_{0}$. The so‐called matrix of regression coefficients $C_{0}{A_{0}}^{-1}$ translates the prior‐to‐posterior change in α to the β scale.

Prior (ii) is a mixture of normal‐inverse‐Wishart distributions. The posterior components also have a closed form. Let μ be partitioned into two vectors corresponding to the observed and unobserved means, α of length J and β of length K. Let Σ be the matrix of covariance parameters conformably partitioned into blocks, $\Sigma =(\begin{array}{cc} A & B\\ C & D \end{array})$. Define a normal‐inverse‐Wishart prior distribution on (μ,Σ) such that $p\left(\mu ,\Sigma \right)=\varphi \left(\mu ;\mu_{0},\Sigma /\lambda_{0}\right)\omega \left(\Sigma ;\Psi_{0},\nu_{0}\right)=\varphi \left((\begin{array}{c} \alpha \\ \beta \end{array});(\begin{array}{c} \alpha_{0}\\ \beta_{0} \end{array}),(\begin{array}{cc} A & B\\ C & D \end{array})/\lambda_{0}\right)\omega \left((\begin{array}{cc} A & B\\ C & D \end{array});(\begin{array}{cc} A_{0} & B_{0}\\ C_{0} & D_{0} \end{array}),\nu_{0}\right)$. Define also $S=D-CA^{-1}B$, and $S_{0}=D_{0}-C_{0}{A_{0}}^{-1}B_{0}$ the Schur complements of A in Σ and $A_{0}$ in $\Psi_{0}$, respectively. Then the posterior can be written as follows:

(9) $$\begin{array}{c} \begin{array}{cc} & p\left(\mu ,\Sigma |x\right)\propto \varphi \left(\alpha ;{\alpha_{0}}^{\prime },\frac{A}{\lambda_{0}^{\prime }}\right)\omega \left(A;{A_{0}}^{\prime },\nu_{0}^{\prime }\right)\\ & \;\varphi \left(\beta ;\beta_{0}+CA^{-1}\left(\alpha -\alpha_{0}\right),\frac{S}{\lambda_{0}}\right)\omega \left(S;S_{0},\nu_{0}\right)\\ & {\left|A\right|}^{-\frac{K}{2}}{\left|S\right|}^{-\frac{J}{2}}\exp\left[-\frac{1}{2}\mathrm{tr}\left(\left(CA^{-1}-C_{0}{A_{0}}^{-1}\right)A_{0}{\left(CA^{-1}-C_{0}{A_{0}}^{-1}\right)}^{T}S^{-1}\right)\right], \end{array} \end{array}$$

where primes denote the usual posterior hyperparameters ${\alpha_{0}}^{\prime }=\left(\lambda_{0}\alpha_{0}+n\bar{x}\right)/\left(\lambda_{0}+n\right)$, ${A_{0}}^{\prime }=A_{0}+\left[\sum_{1}^{n}\left(x_{i}-\bar{x}\right){\left(x_{i}-\bar{x}\right)}^{T}\right]+\left[\lambda_{0}n/\left(\lambda_{0}+n\right)\right]\left(\alpha_{0}-\bar{x}\right){\left(\alpha_{0}-\bar{x}\right)}^{T}$, $\lambda_{0}^{\prime }=\lambda_{0}+n$ and $\nu_{0}^{\prime }=\nu_{0}+n$. The posterior distribution is not a normal‐inverse‐Wishart distribution, it does, however, contain the product of a normal‐inverse‐Wishart density function on the observed visits with updated degrees of freedom, location, and scale parameters, with a normal‐inverse‐Wishart density function of the means and Schur complement of the covariance matrix at the unobserved visits with the same degrees of freedom as the prior. The Schur complement of A is a function of the covariance matrix at unobserved visits, so if the prior degrees of freedom $\nu_{0}$ are sufficiently small for the prior mean of the unobserved visits β to exhibit Cauchy heavy tails then the updated posterior mean of the unobserved visits will continue to exhibit these Cauchy tails. When K=0, that is, all visits are observed, the density function is the usual updated normal‐inverse‐Wishart posterior distribution and when J=0, that is, there are no observed visits, the density function reduces to the normal‐inverse‐Wishart prior distribution

Regardless of the dimension of the current data, the posterior components from prior (iii) have no closed‐form and have to be sampled using an MCMC algorithm.

### 3.4 Posterior Weights

The weight on each component in the mixture posterior is proportional to the marginal likelihood of the data multiplied by the prior mixture weight (Bernardo and Smith 2000). The marginal likelihoods, and so also the posterior weights for priors (i) and (ii), have a closed form. For the full derivation, see the Supporting Information. Let $\alpha_{\inf}$ and $\alpha_{\mathrm{vag}}$ be subvectors, of dimension J, of $\mu_{\inf}$ and $\mu_{\mathrm{vag}}$ and let $A_{\inf}$ and $A_{\mathrm{vag}}$, be submatrices, of dimension J×J, of $\Sigma_{\inf}$ and $\Sigma_{\mathrm{vag}}$, which correspond to the parameters in the multivariate normal mixture prior on the mean for the observed current data. The posterior weight on the informative component in prior (i) is given by

(10) $$\begin{array}{cc} {\hat{w}}_{\text{inf}}^{\prime } & =\frac{w_{\text{inf}}\varphi \left(\bar{x};\alpha_{\inf},A_{\inf}+\frac{1}{n}\hat{\Sigma }\right)}{w_{\text{inf}}\varphi \left(\bar{x};\alpha_{\inf},A_{\inf}+\frac{1}{n}\hat{\Sigma }\right)+w_{\text{vag}}\varphi \left(\bar{x};\alpha_{\mathrm{vag}},A_{\mathrm{vag}}+\frac{1}{n}\hat{\Sigma }\right)}. \end{array}$$

Let $\alpha_{\inf}$ and $\alpha_{\mathrm{vag}}$ be subvectors, of dimension J, of $\mu_{\inf}$ and $\mu_{\mathrm{vag}}$, and let $A_{\inf}$ and $A_{\mathrm{vag}}$ be submatrices, of dimension J×J, of $\Psi_{\inf}$ and $\Psi_{\mathrm{vag}}$ which correspond to the parameters in the normal‐inverse‐Wishart joint mixture prior on the mean and variance for the observed current data. The posterior weight on the informative component for prior (ii) is given as follows:

(11) winf′=winfλinf′λinf−J2ΓJνinf′−K2Ainfνinf−K2ΓJνinf−K2Ainf′νinf′−K2winfλinf′λinf−J2ΓJνinf′−K2Ainfνinf−K2ΓJνinf−K2Ainf′νinf′−K2+wvagλvag′λvag−J2ΓJνvag′−K2Avagνvag−K2ΓJνvag−K2Avag′νvag′−K2.

When the prior and the analysis models share a common dimension J, that is, K=0, the weights in (10) and (11) simplify. The vectors $\alpha_{\inf}$ and $\alpha_{\mathrm{vag}}$ become $\mu_{\inf}$ and $\mu_{\mathrm{vag}}$, respectively, and the matrices $A_{\inf}$, $A_{\mathrm{vag}}$, $A_{\inf}^{\prime }$, and $A_{\mathrm{vag}}^{\prime }$ become $\Sigma_{\inf}$, $\Sigma_{\mathrm{vag}}$, $\Sigma_{\inf}^{\prime }$, and $\Sigma_{\mathrm{vag}}^{\prime }$, respectively. If one chooses to assign equipoise prior weights to the informative and vague components ($w_{\text{inf}}=w_{\text{vag}}=0.5$), this leads to the prior weights cancelling. The posterior weights can then be interpreted as the probability (restricted to the available choice of priors) that the current data were generated by that prior process based solely on the evidence from the data.

### 3.5 Effective Sample Size of a Multivariate Mixture Prior

The calculation of effective sample size (ESS), particularly in a multivariate setting, is not trivial. For a univariate normal likelihood and conjugate normal prior, the variance ratio ESS of Pennello and Thompson (2007) is easy to calculate and equal to the predictively consistent expected local‐information‐ratio ESS described by Neuenschwander et al. (2020). We therefore use the inverse relationship between *total* sample size and the variance of the posterior distribution for a mean parameter to calculate the ESS contributed by a multivariate prior distribution for a marginal univariate posterior distribution. In univariate analyses, the effective sample size of the data, ${\text{ESS}}_{\text{data}}$ is equal to the number of participants in the study, ${\text{ESS}}_{\text{data}}=n$. In the multivariate analysis of data from J visits and a prior based on J+K visits, the ESS of the data on the marginal posterior of the mean at say the J+Kth visit (or any visit for which data is not observed) is less obvious, and not simply equal to n. A value for ${\text{ESS}}_{\text{data}}$ can, however, be found using the variance of two posteriors for which the ESS of the prior is known. Two such posteriors are available, the first is found using just the informative component constructed from $n_{0}$ historical controls as a prior, and the second is found using just the vague component constructed from one representative historical control as a prior. The ${\text{ESS}}_{\text{data}}$ can then be found using the following relationship:

$$\begin{array}{c} \frac{n_{0}+{\text{ESS}}_{\text{data}}}{1+{\text{ESS}}_{\text{data}}}=\frac{\text{Var}(\mu |x,\text{vague prior})}{\text{Var}(\mu |x,\text{informative prior})}. \end{array}$$

Having found ${\text{ESS}}_{\text{data}}$, the variance ratio relationship can be used again to find the ESS of the mixture prior, denoted ${\text{ESS}}_{\text{prior}}$ as follows:

$$\begin{array}{c} \frac{{\text{ESS}}_{\text{prior}}+{\text{ESS}}_{\text{data}}}{1+{\text{ESS}}_{\text{data}}}=\frac{\text{Var}(\mu |x,\text{vague prior})}{\text{Var}(\mu |x,\text{mixture prior})}. \end{array}$$

## 4 Results

### 4.1 Dynamic Borrowing Example With Multivariate Normal Data

In this illustrative example, we consider a model of data at two visits so that the multivariate distributions of the mean parameters can be displayed as a heat map. The priors are defined on J+K=2 dimensions. To fit prior (i), we assumed that an informative multivariate normal distribution component on mean outcomes came from a historical study with $n_{0}=20$ and constructed a vague multivariate normal component by scaling the covariance matrix of the informative component such that it contained the information associated with just a single representative participant. The hyperparameters used are as follows:

$$\begin{array}{cccc} \mu_{\inf}=\left(\begin{array}{c} 5\\ 5 \end{array}\right), & \Sigma_{\inf}=\left(\begin{array}{cc} 1 & 0.5\\ 0.5 & 1 \end{array}\right), & \mu_{\mathrm{vag}}=\left(\begin{array}{c} 5\\ 5 \end{array}\right), & \Sigma_{\mathrm{vag}}=\left(\begin{array}{cc} 20 & 10\\ 10 & 20 \end{array}\right). \end{array}$$

For prior (ii), we assumed a normal‐inverse‐Wishart distribution with a vague marginal distribution on the covariance matrix for both the informative and vague components. The $\lambda_{*}$ hyperparameters control the precision of the prior on the mean parameters. By setting $\nu_{*}=2$, marginally, at each visit, the mean parameters in each component are t‐distributed with one degree of freedom (i.e., Cauchy distributed) with the same location and scale hyperparameters as for priors (i) and (iii),

$$\begin{array}{ccc} \mu_{\inf} & = & \mu_{\mathrm{vag}}=\left(\begin{array}{c} 5\\ 5 \end{array}\right),\lambda_{\text{inf}}=20,\lambda_{\text{vag}}=1,\Psi_{\inf}=\Psi_{\mathrm{vag}}=\left(\begin{array}{cc} 20 & 10\\ 10 & 20 \end{array}\right),\\ \nu_{\text{inf}} & = & \nu_{\text{vag}}=2. \end{array}$$

**FIGURE 2:**
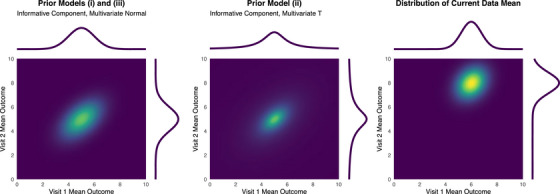
Marginal mean informative density functions and current data mean density function.

For prior (iii), we fitted a mixture of product priors with the same location hyperparameters in the mean density function as for prior (i), and the same scale hyperparameters in the covariance density function as for prior (ii). For all priors, we specified $w_{\text{inf}}=w_{\text{vag}}=0.5$ equipoise weights on these components. The marginal informative components on the mean parameters for all the priors are shown in Figure 2. The code for fitting the illustrative example with the RStan package (Stan Development Team 2025) in R (R Core Team 2025) and with the MCMC procedure in the SAS/STAT software is provided in the Supporting Information.

#### 4.1.1 Full Current Study Data

First, consider a current study with both visits observed so that J=2 and K=0. A single sample with n=30 of the current study control data is described by the sample mean and sample covariance,

$$\begin{array}{c} \bar{x}=\left(\begin{array}{c} 6\\ 8 \end{array}\right),\hat{\Sigma }=\left(\begin{array}{cc} 18 & 5\\ 5 & 22 \end{array}\right) \end{array}.$$

**FIGURE 3:**
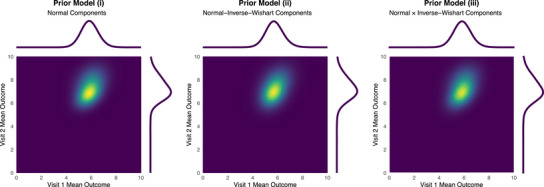
Marginal posterior distributions of the mean parameters from two‐dimensional sample data.

The estimated bivariate mean from the current study data is plotted in Figure 2. The density is shown on the same scale as the priors to enable a comparison of the relative weight of evidence in the prior–data combination. Priors (i) and (ii) are conjugate to a multivariate normal likelihood function, and so the resulting posteriors can be written down explicitly. The posterior resulting from prior (i) is a mixture of multivariate normal distributions given by (7) or (8) weighted according to (10). The posterior resulting from prior (ii) is a mixture of normal‐inverse‐Wishart distributions given by (9) weighted according to (11). The posterior resulting from prior (iii) has to be sampled. To do this, we created and analyzed a sample with empirical summary statistics matching those given above. The posterior distributions for the mean parameters are plotted in Figure 3. The posterior density functions and equal‐tailed credible intervals are given in Table 1. The posterior credible intervals are broadly similar, with those resulting from prior (i), which ignores uncertainty in the covariance estimate, unsurprisingly, the narrowest. The heavier tails of the informative component in prior (ii) allow more of the posterior weight to be assigned to the posterior component associated with the informative prior, but that gain in borrowing largely cancels out with the loss in precision from modeling the covariance. The marginal posterior distributions of the mean parameters arising from prior (iii) have heavier tails than those arising from prior (ii). A prior constructed as a product of independent distributions of the mean and covariance parameters does not correctly describe the sampling distribution from the historical data because the sample mean and covariance are necessarily corrected. Unlike the normal‐inverse‐Wishart distributions used in prior (ii), the product prior does not describe that correlation.

**TABLE 1:** Marginal posterior properties of the mean parameters from two‐dimensional sample data.

	Prior model (i)	Prior model (ii)	Prior model (iii)
Density function	$$\begin{array}{c} 0.52\varphi \left(\left(\begin{array}{c} 5.77\\ 6.72 \end{array}\right),\left(\begin{array}{c} 0.370.14\\ 0.140.41 \end{array}\right)\right)\\ +0.48\varphi \left(\left(\begin{array}{c} 5.99\\ 7.89 \end{array}\right),\left(\begin{array}{c} 0.580.17\\ 0.170.71 \end{array}\right)\right) \end{array}$$	$$\begin{array}{c} 0.58t_{\text{df=31}}\left(\left(\begin{array}{c} 5.60\\ 6.80 \end{array}\right),\left(\begin{array}{c} 0.370.13\\ 0.130.51 \end{array}\right)\right)\\ +0.42t_{\text{df=31}}\left(\left(\begin{array}{c} 5.97\\ 7.90 \end{array}\right),\left(\begin{array}{c} 0.580.17\\ 0.170.72 \end{array}\right)\right) \end{array}$$	—
**Visit 1**			
Mean (95% credible interval)	5.88(4.54,7.28)	5.76(4.40,7.23)	5.86(4.47,7.31)
**Visit 2**			
Mean (95% credible interval)	7.28(5.63,9.25)	7.28(5.54,9.26)	7.23(5.46,9.27)

#### 4.1.2 Partial Current Study Data

**FIGURE 4:**
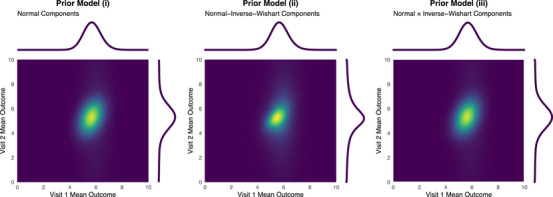
Marginal posterior distributions of the mean parameters from one‐dimensional sample data.

The example can be repeated, reducing the dimensions of the current study data so that only the first visit is observed, that is, J=1 and K=1. Take the observed data to be the Visit 1 sample from Section 4.1.1 with $\bar{x}=6$, $\hat{\Sigma }=18$ from n=30. The posterior density functions resulting from priors (i) and (ii) can be written down using the components described in (7) or (8) and (9), respectively, and the posterior weights from (10) and (11), respectively. The posterior resulting from prior (iii) must again be sampled. The posterior distributions for the mean parameters are plotted in Figure 4, and the posterior density functions and equal‐tailed credible intervals are given in Table 2. The posterior distribution on the mean parameter at the unobserved visit resulting from prior (ii) exhibits Cauchy‐like behavior since both components of the prior used degrees of freedom $\nu_{\text{inf}}=\nu_{\text{vag}}=2$. The mean parameters are shifted by the observed data at Visit 1 due to the correlation in the prior, but, since no data were observed, the degrees of freedom in the posterior Wishart density functions are unchanged. In all the models, a higher posterior weight is assigned to the informative component than to the equivalent model in Section 4.1.1. This is because of the relatively greater similarity of the current study data to the informative component at the first visit than at the second visit. As a result, despite no study data being observed at the second visit, the credible interval for the second visit mean parameter is relatively narrow. The assumed correlation between mean outcomes pulls the posterior mean at the second visit higher than the prior mean. As before, the marginal mean posterior distributions resulting from priors (ii) and (iii) have heavier tails than the posterior resulting from prior (i).

**TABLE 2:** Marginal posterior properties of the mean parameters from one‐dimensional sample data.

	Prior model (i)	Prior model (ii)	Prior model (iii)
Density function	$$\begin{array}{c} 0.73\varphi \left(\left(\begin{array}{c} 5.63\\ 5.31 \end{array}\right),\left(\begin{array}{c} 0.380.19\\ 0.190.84 \end{array}\right)\right)\\ +0.27\varphi \left(\left(\begin{array}{c} 5.97\\ 5.49 \end{array}\right),\left(\begin{array}{c} 0.580.29\\ 0.2915.15 \end{array}\right)\right) \end{array}$$	see Equations 9 and 11	—
**Visit 1**			
Mean (95% credible interval)	5.72(4.44,7.10)	5.70(4.39,7.12)	5.71(4.38,7.12)
**Visit 2**			
Mean (95% credible interval)	5.36(0.32,10.65)	5.35(−1.27,12.24)	5.36(0.31,10.66)

There may be a temptation to use a different vague component in the prior, which assumes no correlation between the observed mean outcomes and the unobserved mean outcomes. A diagonal covariance matrix might be thought of as in some way “less informative” because the marginal posterior of the mean at the unobserved second visit will be unaffected by observed data. We suggest that this would be a mistake. The prior assumption of no correlation is very strong and unlikely to be true. The data from one visit should implicitly carry some information about the mean outcome at other visits because of the intrinsic correlation of repeated measures data. To understand the importance of using nonzero correlation values in the formulation of the prior, one could repeat the analysis using diagonal covariance matrix hyperparameters in the prior to observe that the marginal posterior of the mean parameters at the unobserved visit will be equal to the reweighted marginal prior.

### 4.2 Dynamic Borrowing in a Randomized Trial With an Open‐Label Extension

#### 4.2.1 Description of Simulations

The motivating randomized trial (n=150 per arm) and its open‐label extension are described in Section 2. This simulation study examines the estimated difference in mean FVC between an active and control arm at 104 weeks under different historical and current treatment effect scenarios and prior weights. The control arm mean was constructed from a Bayesian dynamic borrowing analysis, which combines the 52‐week current control arm with a 104‐week historical control arm $n_{0}=200$. A posterior treatment effect was constructed from the difference between an active arm and the combined control data. Due to the large sample sizes, we used prior (i) with the known covariance matrix assumption to model the mean outcome. In general, accounting for uncertainty by modeling covariance matrix parameters with priors (ii) or (iii) will reduce the precision of the posterior mean parameter estimates but should be preferred unless the population covariance is somehow known. However, for sufficiently large sample sizes, that is, as a t‐distribution tends to a normal distribution, and for this simulation study, the differences in precision with which the mean parameters can be estimated will be negligible.

A formal prior elicitation (Dallow et al. 2018) was performed when planning the motivating randomized clinical trial and suggested a 52‐week mean treatment effect on FVC of approximately 100 mL, and so the simulations cover a range of treatment effects around this value. Depending on the form of the observed outcome trajectory in the historical controls, the planned analysis may use a by‐visit analysis model, or an intercept–slope analysis model. If the historical control data suggests a linear trajectory then the intercept–slope analysis model will be appropriate. In the intercept–slope analysis model, the prior distribution is bivariate normal on the intercept and slope of a linear model. It is possible to translate between a by‐visit prior on mean outcomes, and a bivariate prior on the mean intercept and slope using the generalized least squares estimator equations (or simulation). Three different priors for the control arm are assessed in the simulation study. First, we assessed a prior on the intercept and slope (milliliter per year) model with $\mu_{\inf}^{\left(1\right)}=\mu_{\mathrm{vag}}^{\left(1\right)}={\left(3000,-100\right)}^{T}$, so the dimension of the prior is J+K=2. Second, we considered an equivalent prior for a by‐visit analysis model with prior means at each visit as follows: $\mu_{\inf}^{\left(2\right)}=\mu_{\mathrm{vag}}^{\left(2\right)}={\left(3000,2975,2950,2925,2900,2875,2850,2825,2800\right)}^{T}$, so the dimension of the prior is J+K=9. Finally, we assessed another by‐visit analysis model with “dog‐leg” prior means, $\mu_{\inf}^{\left(3\right)}=\mu_{\mathrm{vag}}^{\left(3\right)}={\left(3000,2950,2900,2850,2800,2800,2800,2800,2800\right)}^{T}$, so the dimension of the prior is again J+K=9. The covariance matrix hyperparameter used for the informative component for these simulations was estimated from data received from the TransCelerate data sharing scheme. This covariance matrix covered a shorter duration (1 year) than the planned historical data will cover, so for this simulation study we extended the by‐visit informative component covariance matrix $\Sigma_{\inf}^{\mathrm{BV}}$ appropriately to cover nine visits over 2 years,

$$\begin{array}{c} \Sigma_{\inf}^{\mathrm{BV}}=3380\left(\begin{array}{ccccccccc} 1 & 0.98 & 0.97 & 0.96 & 0.95 & 0.94 & 0.93 & 0.92 & 0.91\\ 0.98 & 1 & 0.98 & 0.97 & 0.96 & 0.95 & 0.94 & 0.93 & 0.92\\ 0.97 & 0.98 & 1 & 0.98 & 0.97 & 0.96 & 0.95 & 0.94 & 0.93\\ 0.96 & 0.97 & 0.98 & 1 & 0.98 & 0.97 & 0.96 & 0.95 & 0.94\\ 0.95 & 0.96 & 0.97 & 0.98 & 1 & 0.98 & 0.97 & 0.96 & 0.95\\ 0.94 & 0.95 & 0.96 & 0.97 & 0.98 & 1 & 0.98 & 0.97 & 0.96\\ 0.93 & 0.94 & 0.95 & 0.96 & 0.97 & 0.98 & 1 & 0.98 & 0.97\\ 0.92 & 0.93 & 0.94 & 0.95 & 0.96 & 0.97 & 0.98 & 1 & 0.98\\ 0.91 & 0.92 & 0.93 & 0.94 & 0.95 & 0.96 & 0.97 & 0.98 & 1 \end{array}\right). \end{array}$$

This covariance matrix corresponds to the assumption of a standard deviation in FVC at each visit of approximately 822 mL and hence is equivalent to an expected standard deviation in the distribution of the sample mean outcome (i.e., a standard error) from $n_{0}=200$ in the historical controls of approximately 58 mL. The translated informative component hyperparameter used in the intercept–slope analysis model $\Sigma_{\inf}^{\mathrm{IS}}$ can be written as follows, where the intercept is the first parameter, and slope (milliliter per year) is the second parameter:

$$\begin{array}{c} \Sigma_{\inf}^{\mathrm{IS}}=\left(\begin{array}{cc} 3372 & -144\\ -144 & 144 \end{array}\right). \end{array}$$

For both, the by‐visit and intercept–slope analysis models, the vague component covariance matrix was obtained from the informative component covariance matrix by increasing all the covariances by a factor of $n_{0}$, $\Sigma_{\mathrm{vag}}^{\mathrm{BV}}=n_{0}\Sigma_{\inf}^{\mathrm{BV}}$, $\Sigma_{\mathrm{vag}}^{\mathrm{IS}}=n_{0}\Sigma_{\inf}^{\mathrm{IS}}$ such that the vague prior represented the information from a single representative historical control. All current data, active and control, were simulated using the implied covariance matrix from the historical controls, $\Sigma =n_{0}\Sigma_{\inf}^{\mathrm{BV}}$. The current control arm was simulated for five visits (including baseline) for a single year but hypothesized to continue unobserved in some pattern for four visits during a second year. The intercept–slope prior and likelihood thus had a common dimension J=2, and no dimensions were unobserved, K=0. However, in the by‐visit analysis models, the first J=5 visits were observed and the final K=4 visits were unobserved. The active arm, which had a mean of 3000 mL at all visits in every scenario, was simulated for all nine visits over 2 years.

Table 3 shows the properties of multivariate Bayesian dynamic borrowing analyses for different historical and current data patterns. In every scenario, the average posterior weight on the informative component, the average bias in the control arm estimate at Week 104, the average standard error of that estimate, the average ESS of the prior on the Week 104 control mean, and the power to declare statistical significance, defined as 97.5% of the posterior for the mean difference between active and control being below zero, was calculated from 100,000 simulations. The table is divided into panels, in the left‐hand panels, the prior assessed has a density function given by $p\left(\mu \right)=w_{\text{inf}}\varphi \left(\mu ;\mu_{\inf}^{\left(1\right)},\Sigma_{\inf}^{\mathrm{IS}}\right)+\left(1-w_{\text{inf}}\right)\varphi \left(\mu ;\mu_{\mathrm{vag}}^{\left(1\right)},\Sigma_{\mathrm{vag}}^{\mathrm{IS}}\right)$, in the central panels, the prior has a density function given by $p\left(\mu \right)=w_{\text{inf}}\varphi \left(\mu ;\mu_{\inf}^{\left(2\right)},\Sigma_{\inf}^{\mathrm{BV}}\right)+\left(1-w_{\text{inf}}\right)\varphi \left(\mu ;\mu_{\mathrm{vag}}^{\left(3\right)},\Sigma_{\mathrm{vag}}^{\mathrm{BV}}\right)$, in the right‐hand panels the prior has a density function given by $p\left(\mu \right)=w_{\text{inf}}\varphi \left(\mu ;\mu_{\inf}^{\left(3\right)},\Sigma_{\inf}^{\mathrm{BV}}\right)+\left(1-w_{\text{inf}}\right)\varphi \left(\mu ;\mu_{\mathrm{vag}}^{\left(3\right)},\Sigma_{\mathrm{vag}}^{\mathrm{BV}}\right)$. Results are given for a range of prior weights $w_{\text{inf}}\in \left\{0,0.5,1\right\}$. Results for $w_{\text{inf}}\in \left\{0.25,0.75\right\}$ are given in the Supporting Information. When the prior weight on the informative component was zero, the analysis used the fixed vague prior on the mean parameters, and when the prior weight on the informative component was one, the analysis used the fixed informative prior on the mean parameters. In the first row of panels, the current control arm was simulated to have a mean of 3000 mL at all visits and was assumed to continue with this mean for the second year, that is, a null treatment effect. In the second row of panels, the current control arm mean was simulated to have a linear descent creating treatment effects from 25 to 100 mL after 1 year and from 50 to 200 mL after 2 years. In the third row of panels, the current control arm mean was simulated to have a “dog‐leg” descent creating treatment effects from 50 to 200 mL after 1 year, which were assumed to remain constant during the second. Finally, in the fourth row of panels, the current control arm means followed a “double‐dog‐leg” pattern, with the treatment effect appearing in the middle third of the 2 years with constant means in the first and final thirds of the 2‐year period.

**TABLE 3:** Simulation study results for marginal estimates at week 104.

				Linear prior data and intercept–slope analysis model					Linear prior data and by‐visit analysis model					Dog‐leg prior data and by‐visit analysis model				
				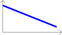					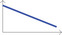					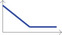				
	Prior	True	True	Average	Average control		Average	Type 1	Average	Average control		Average	Type 1	Average	Average control		Average	Type 1
	weight	active	control	posterior	bias	SE	ESS of	error or	posterior	bias	SE	ESS of	error or	posterior	bias	SE	ESS of	error or
	$w_{\text{inf}}$	mean (mL)	mean (mL)	weight $w_{\text{inf}}^{\prime }$	(mL)	(mL)	prior	power	weight $w_{\text{inf}}^{\prime }$	(mL)	(mL)	prior	power	weight $w_{\text{inf}}^{\prime }$	(mL)	(mL)	prior	power
**Null effect current data**																		
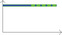	0	3000	3000	0	−3	72	1	0.03	0	−103	239	1	0.00	0	−5	239	1	0.00
	0.5			0.07	−10	73	−4	0.04	0.78	−143	102	87	0.28	0.00	−5	239	1	0.00
	1			1	−146	45	200	0.46	1	−157	45	200	0.50	1	−115	45	200	0.28
**Linear current data**																		
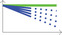	0	3000	2950	0	−2	72	1	0.08	0	−77	239	1	0.00	0	20	239	1	0.00
			2900		−1			0.19		−51			0.00		46			0.00
			2850		−1			0.35		−25			0.00		71			0.00
			2800		0†			0.54		0†			0.00		98			0.00
	0.5	3000	2950	0.33	−29	72	9	0.16	0.96	−116	57	159	0.50	0.01	20	238	1	0.00
			2900	0.70	−44	61	73	0.39	>0.99	−79	46	192	0.61	0.09	40	227	4	0.01
			2850	0.91	−31	50	146	0.60	>0.99	−39	45	198	0.67	0.39	48	178	24	0.07
			2800	0.96	0†	47	172	0.71	>0.99	0†	45	199	0.72	0.78	57	102	87	0.28
	1	3000	2950	1	−110	45	200	0.52	1	−118	45	200	0.56	1	−75	45	200	0.33
			2900		−73			0.60		−79			0.61		−36			0.38
			2850		−37			0.67		−39			0.67		3			0.44
			2800		0†			0.73		0†			0.72		43			0.49
**Dog‐leg current data**																		
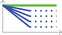	0	3000	2950	0	−51	72	1	0.19	0	−101	239	0	0.00	0	−4	239	1	0.00
			2900		−100‡			0.54		−100‡			0.00		−3			0.00
			2850		−149			0.86		−99			0.01		−1			0.00
			2800		−197			0.98		−97			0.02		0†			0.00
	0.5	3000	2950	0.70	−94	61	73	0.39	>0.99	−128	47	192	0.61	0.09	−10	227	4	0.01
			2900	0.96	−100‡	47	172	0.71	>0.99	−100‡	45	199	0.72	0.78	−43	102	87	0.28
			2850	0.70	−106	61	73	0.85	>0.99	−71	46	192	0.80	>0.99	−29	46	192	0.61
			2800	0.07	−190	73	−4	0.98	0.78	−58	103	87	0.62	>0.99	0†	45	199	0.72
	1	3000	2950	1	−123	45	200	0.60	1	−129	45	200	0.61	1	−86	45	200	0.38
			2900		−100‡			0.72		−100‡			0.72		−57			0.50
			2850		−77			0.83		−71			0.81		−29			0.61
			2800		−54			0.91		−43			0.88		0†			0.72
**Double‐dog‐leg current data**																		
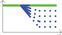	0	3000	2950	0	−8	72	1	0.09	0	−77	239	1	0.00	0	21	239	1	0.00
			2900		−12			0.22		−52			0.00		46			0.00
			2850		−16			0.41		−26			0.00		72			0.00
			2800		−21			0.62		0			0.00		97			0.00
	0.5	3000	2950	0.38	−38	71	14	0.18	0.95	−115	60	152	0.48	0.01	20	238	1	0.00
			2900	0.78	−55	58	94	0.44	0.96	−77	57	159	0.55	0.05	42	232	2	0.00
			2850	0.94	−37	49	158	0.64	0.85	−37	85	109	0.47	0.11	65	224	5	0.01
			2800	0.93	−7	49	157	0.73	0.41	0	174	27	0.15	0.09	94	227	4	0.01
	1	3000	2950	1	−111	45	200	0.53	1	−118	45	200	0.56	1	−75	45	200	0.33
			2900		−76			0.61		−79			0.61		−36			0.38
			2850		−40			0.68		−39			0.67		3			0.44
			2800		−5			0.74		0			0.72		43			0.50

† No prior‐current data conflict.

‡ No short‐term prior‐current data conflict followed by unobserved long‐term conflict.

#### 4.2.2 Simulation Results

Each panel in Table 3 can be understood as a separate simulation study. The top three panels show a null treatment effect. The bottom nine panels show treatment effects in the current data, which vary from 50 to 200 mL after 2 years. Reading down the panels, one can see the properties of an analysis on different current data but always using the same historical data. Comparing across the panels, one can see the properties of using different priors derived from historical data on the same current data. In particular, comparing across the panels in the first two columns, one can understand the difference between an intercept–slope modeling assumption and a by‐visit modeling assumption. Simulations in which the 2‐year historical control and the 1‐year current control arm participants are exchangeable, that is, in which there is no prior‐current data conflict are marked in the table with a dagger. In all cases, the bias in the estimate of the control outcome is zero. Unbiased estimates of the control outcome can be used to produce causally valid, long‐term treatment effect estimates. Simulations in which there is no short‐term prior‐current data conflict, followed by conflict are marked with a double dagger. In all these simulations, the posterior borrowing from the informative component is high but the assumed trajectory of the unobserved current data diverges from the prior, and so bias is seen. In all the other simulations, there is some form of prior‐current data conflict between the historical and current control arm. In each panel of simulations, the posterior borrowing can be seen to increase as the prior weight on the informative component increases and as the current control arm better matches the historical control. The ${\text{ESS}}_{\text{prior}}$ on the control mean estimate at Week 104 increases from 1 to 200 as the posterior weight increases from 0 to 1. A negative ${\text{ESS}}_{\text{prior}}$ can be seen when the standard error of the control arm estimate from an analysis with a robust mixture prior is greater than the standard error from an analysis with the fixed vague prior. In addition to the results in the table, the average ${\text{ESS}}_{\text{data}}$ values on the control mean estimate at Week 104 reveal the effects of the different analysis model assumptions on the posterior estimate at the unobserved Week 104 visit. In all the intercept–slope analysis model scenarios, the average ${\text{ESS}}_{\text{data}}$ was approximately 127. That is, with an intercept–slope model and a corresponding prior, the statistical information from 200 participants at the first five visits was equivalent to having 127 participants at the ninth visit. In all the by‐visit analysis model scenarios, the average ${\text{ESS}}_{\text{data}}$ was approximately 6.3. That is, with a by‐visit model and a corresponding prior, the statistical information from 200 participants at the first five visits was equivalent to having 6.3 participants at the ninth visit. So even with the high between‐visit correlation coefficients used in this example, the constant slope assumption “transmits” far more statistical information to the Week 104 control mean estimate than a covariance matrix.

In the left‐hand panels, using equipoise prior weights in an intercept–slope analysis model, the robust mixture prior maintains a type 1 error rate of 0.04. The power increase from a robust mixture prior compared with a fixed vague prior can be substantial; the largest increase is 25 percentage points from 35% to 60%. Two scenarios show a decrease in power from 98% to 97% and 86% to 85%. In the central panels, using equipoise prior weights in a by‐visit analysis model, the robust mixture prior has a 28% type one error for the null scenario considered. This reflects the more relaxed assumptions of the model regarding linearity. The five control mean values in the current data are 0, 25, 50, 75, and 100 mL, different from the historical control means, and these differences are not large enough to prevent high posterior borrowing. In terms of the control of type one error, the intercept–slope modeling assumption appears preferable. The right‐hand panels show the results from a differently shaped prior distribution, which, because it is not linear, should logically be combined with the by‐visit modeling assumption. In this case, the type one error with a robust mixture prior is well controlled at less than 1%. The increases in power compared with a fixed vague prior are sometimes substantial, in one case from less than 1% power to 72% power.

## 5 Discussion

We have used Bayes' theorem to derive posterior distributions from multivariate mixture priors with a possibly reduced dimension multivariate normal likelihood function. Specifically, when the prior is a mixture of multivariate normal distributions on a mean parameter vector; or when the prior is a mixture of normal‐inverse‐Wishart distributions on the mean parameter vector and a matrix of covariance parameters. The closed‐form posteriors enable Bayesian dynamic borrowing analyses to be performed easily on multivariate data without even the need for individual participant data, so the method can be applied to published results or using summary statistics provided by third parties. The code is provided in the Supporting Information for specifying more general nonconjugate prior distributions and sampling the resulting posteriors. At unobserved visits, the posteriors describe the distribution of the treatment effect given a prior assumption and partially observed data, essentially solving a missing data problem by incorporating a prior belief.

The analyses described in this paper are of continuous data, but the method is more generally applicable to any parameter model which can be modeled with a multivariate normal likelihood function. For example, piecewise linear models of continuous data, or repeated and correlated log‐odds or log‐hazard parameters for binary or time‐to‐event data. Aggregate statistics can be modeled directly with a multivariate normal likelihood if, say, the sample sizes are large enough for the central limit theorem to hold.

Multivariate Bayesian dynamic borrowing is also perfectly amenable to borrowing directly on the treatment effect. For example, using the treatment effect from adult studies when extrapolating to a pediatric population. We have described borrowing on a single clinical outcome with repeated measures, but there are potential applications of the method in modeling joint outcomes cross‐sectionally or longitudinally. For example, in oncology trials, updating a joint progression‐free survival, overall survival prior to using available early phase progression‐free survival data for decision‐making based on the marginal overall survival posterior.

Through simulation, it has been demonstrated that the method can be used to produce unbiased and hence causally valid estimates of long‐term treatment effects from a single active‐arm open‐label extension following a randomized clinical trial when the current participants and the historical participants are exchangeable. The results also show scenarios with prior‐current data conflict and show that bias in estimates can arise. However, quantifying the bias from analyzing partially unobserved current data is quite a limited way to understand the methodology. It is possible to create bias of an arbitrary magnitude by changing the trajectory of the control arm from the current data after the current controls have ceased to be followed up, and this does not really reflect a failing of Bayesian dynamic borrowing, but rather reflects the limitations of analyzing current control data with a short follow‐up. Using informative priors does not guarantee type 1 error control. If the informative prior belief about the control arm is not precise enough to exclude a trajectory resembling the active arm, there is potential for type 1 error inflation. In the planning phase, the potential for type 1 error inflation should be assessed for a range of null hypotheses. A slowly emerging prior‐current data conflict may be difficult to pick up from short‐term current data. In this case, the prior weights can be specified to better control the type 1 error by reducing the prior weight given to the informative component, forcing the study data to resemble the informative component much more closely before strong borrowing occurs. In some situations, intercept–slope analysis models may pick up this early conflict more readily and might be preferable if the data support a constant slope model.

Borrowing information on a continuous endpoint can be performed on various metrics, for example, the outcome value, the change from baseline, or a slope. Each approach implies quite different borrowing behavior. Constant slope models inherently already “borrow” substantially from early timepoints, and so even if a prior‐current data conflict exists, the posterior will be relatively narrow without borrowing from historical data. Borrowing on the change from baseline value implies that the starting, baseline level is not relevant to whether the prior‐current data conflict exists, and only the post‐baseline trend matters. Borrowing on the absolute value is more constraining. These differences are important to the analysis and should be understood and discussed when planning. The open‐label extension data from control‐arm randomized controlled trial participants who switch to active treatment could, in principle, be used to help estimate active arm outcomes, perhaps after recentering either the time at baseline or recentering the control arm outcomes at the active arm mean at entry to the open‐label extension.

By borrowing historical data according to commensurability with the current randomized trial data, the targeted estimand population is that of the randomized trial. However, the method does not address bias in estimates arising from missing not‐at‐random data during the trial. These estimation issues arise in all clinical trial analyses and continue to exist in analyses with dynamic borrowing. Estimates are unbiased only when the historical participants and current participants are exchangeable, so every effort should be made to ensure exchangeability before applying Bayesian dynamic borrowing. Exchangeability can, for example, be assessed by referring to the criteria laid out in Pocock (1976). Collecting data contemporaneously can ensure that these criteria are more likely to be met, in particular, with regard to participants being treated with the same methods, and ensuring participants are part of a study with the same eligibility criteria.

## Conflicts of Interest

BH is a contractor working for GSK. MP and AM are employees of and hold shares in GSK.

## Open Research Badges

This article has earned an Open Data badge for making publicly available the digitally‐shareable data necessary to reproduce the reported results. The data is available in the Supporting Information section.

This article has earned an open data badge “**Reproducible Research**” for making publicly available the code necessary to reproduce the reported results. The results reported in this article could fully be reproduced.

## Supporting information


**Supporting file 1:** bimj70079‐sup‐0001‐SuppMat.pdf

**Supporting file 2:** bimj70079‐sup‐0002‐Datacode.zip

## Data Availability

Simulation code is provided. Software in the form of SAS and R code is available in the Supporting Information. SAS and all other SAS Institute Inc. product or service names are registered trademarks or trademarks of SAS Institute Inc. in the USA and other countries. indicates USA registration.

## References

[bimj70079-bib-0001] Bennett, M. , S. White , N. Best , and A. Mander . 2021. “A Novel Equivalence Probability Weighted Power Prior for Using Historical Control Data in an Adaptive Clinical Trial Design: A Comparison to Standard Methods.” Pharmaceutical Statistics 20, no. 3: 462–484. 33474798 10.1002/pst.2088 PMC8611797

[bimj70079-bib-0002] Bernardo, J. M. , and A. Smith . 2000. Bayesian Theory. John Wiley & Sons Ltd.

[bimj70079-bib-0003] Best, N. , M. Ajimi , B. Neuenschwander , G. Saint‐Hilary , and S. Wandel . 2024. “Beyond the Classical Type I Error: Bayesian Metrics for Bayesian Designs Using Informative Priors.” Statistics in Biopharmaceutical Research, 17, no. 2: 183–196. 10.1080/19466315.2024.2342817.

[bimj70079-bib-0004] Callegaro, A. , N. Galwey , and J. J. Abellan . 2023. “Historical Controls in Clinical Trials: A Note on Linking Pocock's Model With the Robust Mixture Priors.” Biostatistics 24: 443–448. 37057610 10.1093/biostatistics/kxab040

[bimj70079-bib-0005] Dallow, N. , N. Best , and T. H. Montague . 2018. “Better Decision Making in Drug Development through Adoption of Formal Prior Elicitation.” Pharmaceutical Statistics 17, no. 4: 301–316. 29603614 10.1002/pst.1854

[bimj70079-bib-0006] Dawid, A. P. 1973. “Posterior Expectations for Large Observations.” Biometrika 60, no. 3: 664–667.

[bimj70079-bib-0007] Duan, Y. , K. Ye , and E. P. Smith . 2006. “Evaluating Water Quality Using Power Priors to Incorporate Historical Information.” Environmetrics 17, no. 1: 95–106.

[bimj70079-bib-0008] FDA . 2020. “Interacting With the FDA on Complex Innovative Trial Designs for Drugs and Biological Products: Guidance for Industry.” FDA‐2019‐D‐3679. Food and Drug Administration.

[bimj70079-bib-0009] Gelman, A. , J. B. Carlin , H. S. Stern , and D. B. Rubin . 1995. Bayesian Data Analysis. Chapman and Hall/CRC.

[bimj70079-bib-0010] Goring, S. , A. Taylor , K. Müller , et al. 2019. “Characteristics of Non‐Randomised Studies Using Comparisons With External Controls Submitted for Regulatory Approval in the USA and Europe: A Systematic Review.” BMJ Open 9, no. 2: e024895. 10.1136/bmjopen-2018-024895 PMC6398650 30819708

[bimj70079-bib-0011] Hobbs, B. P. , B. P. Carlin , S. J. Mandrekar , and D. J. Sargent . 2011. “Hierarchical Commensurate and Power Prior Models for Adaptive Incorporation of Historical Information in Clinical Trials.” Biometrics 67: 1047–1056. 21361892 10.1111/j.1541-0420.2011.01564.x PMC3134568

[bimj70079-bib-0012] Ibrahim, J. G. , and M.‐H. Chen . 2000. “Power Prior Distributions for Regression Models.” Statistical Science 15: 46–60.

[bimj70079-bib-0013] ICH . 2020. “ICH E9 (R1) Addendum on Estimands and Sensitivity Analysis in Clinical Trials to the Guideline on Statistical Principles for Clinical Trials.” EMA/CHMP/ICH/436221/2017. The International Council for Harmonisation of Technical Requirements for Pharmaceuticals for Human Use.

[bimj70079-bib-0014] Lin, J. , M. Gamalo‐Siebers , and R. Tiwari . 2019. “Propensity‐Score‐Based Priors for Bayesian Augmented Control Design.” Pharmaceutical Statistics 18, no. 2: 223–238. 30537087 10.1002/pst.1918

[bimj70079-bib-0015] Lindley, D. V. 1968. “The Choice of Variables in Multiple Regression.” Journal of the Royal Statistical Society, Series B: Statistical Methodology 30, no. 1: 31–53.

[bimj70079-bib-0016] Megan, B. , R. M. Pickering , and M. Weatherall . 2012. “Design, Objectives, Execution and Reporting of Published Open‐Label Extension Studies.” Journal of Evaluation in Clinical Practice 18, no. 2: 209–215. 21040252 10.1111/j.1365-2753.2010.01553.x

[bimj70079-bib-0017] Neuenschwander, B. , M. Branson , and D. J. Spiegelhalter . 2009. “A Note on the Power Prior.” Statistics in Medicine 28: 3562–3566. 19735071 10.1002/sim.3722

[bimj70079-bib-0018] Neuenschwander, B. , G. Capkun‐Niggli , M. Branson , and D. J. Spiegelhalter . 2010. “Summarizing Historical Information on Controls in Clinical Trials.” Clinical Trials 7, no. 1: 5–18. 20156954 10.1177/1740774509356002

[bimj70079-bib-0019] Neuenschwander, B. , S. Weber , H. Schmidli , and A. O'Hagan . 2020. “Predictively Consistent Prior Effective Sample Sizes.” Biometrics 76, no. 2: 578–587. 32142163 10.1111/biom.13252

[bimj70079-bib-0020] O'Hagan, A. 1979. “On Outlier Rejection Phenomena in Bayes Inference.” Journal of the Royal Statistical Society, Series B: Statistical Methodology 41, no. 3: 358–367.

[bimj70079-bib-0021] Pennello, G. , and L. Thompson . 2007. “Experience With Reviewing Bayesian Medical Device Trials.” Journal of Biopharmaceutical Statistics 18, no. 1: 81–115. 10.1080/10543400701668274 18161543

[bimj70079-bib-0022] Pocock, S. J. 1976. “The Combination of Randomized and Historical Controls in Clinical Trials.” Journal of Chronic Diseases 29: 175–188. 770493 10.1016/0021-9681(76)90044-8

[bimj70079-bib-0023] Psioda, M. A. , and J. G. Ibrahim . 2019. “Bayesian Clinical Trial Design Using Historical Data That Inform the Treatment Effect.” Biostatistics 20, no. 3: 400–415. 29547966 10.1093/biostatistics/kxy009 PMC6587921

[bimj70079-bib-0024] R Core Team . 2025. R: A Language and Environment for Statistical Computing . R Foundation for Statistical Computing. Version 4.5.0 (2025‐04‐11).

[bimj70079-bib-0025] Schmidli, H. , S. Gsteiger , S. Roychoudhury , A. O'Hagan , D. Spiegelhalter , and B. Neuenschwander . 2014. “Robust Meta‐Analytic‐Predictive Priors in Clinical Trials With Historical Control Information.” Biometrics 70: 1023–1032. 25355546 10.1111/biom.12242

[bimj70079-bib-0026] Spiegelhalter, D. J. , K. R. Abrams , and J. P. Myles . 2004. Bayesian Approaches To Clinical Trials and Health‐Care Evaluation. Statistics in Practice, Vol. 13. John Wiley & Sons.

[bimj70079-bib-0027] Stan Development Team . 2025. “RStan: The R Interface to Stan.” R Package Version 2.32.7.

[bimj70079-bib-0028] Taylor, G. J. , and P. Wainwright . 2005. “Open Label Extension Studies: Research or Marketing?” BMJ 331, no. 7516: 572–574. 16150772 10.1136/bmj.331.7516.572 PMC1200598

[bimj70079-bib-0029] Viele, K. , S. Berry , B. Neuenschwander . et al. 2014. “Use of Historical Control Data for Assessing Treatment Effects in Clinical Trials.” Pharmaceutical Statistics 13, no. 1: 41–54. 23913901 10.1002/pst.1589 PMC3951812

[bimj70079-bib-0030] Wang, C.‐Y. , J. A. Berlin , B. Gertz . et al. 2022. “Uncontrolled Extensions of Clinical Trials and the Use of External Controls—Scoping Opportunities and Methods.” Clinical Pharmacology & Therapeutics 111, no. 1: 187–199. 34165790 10.1002/cpt.2346 PMC9290853

