# Supplementary material for: Multivariate Bayesian Dynamic Borrowing for Repeated Measures Data With Application to External Control Arms in Open‐Label Extension Studies
Source: Biom J. 2025 Oct 7;67(5):e70079. doi: 10.1002/bimj.70079 (PMC12504158; doi:10.1002/bimj.70079)
Supplement: Supplementary file 1 — Supporting file 1: bimj70079‐sup‐0001‐SuppMat.pdf [file BIMJ-67-e70079-s001.pdf]

Biometrical Journal **52** (2010) 61, zzz–zzz / DOI: 10.1002/bimj.200100000

Supplementary Information for Multivariate Bayesian dynamic borrowing for repeated measures data with application to external control arms in open-label extension studies

Benjamin F. Hartley*[1], **Matthew A. Psioda**[2], and **Adrian P. Mander**[3]

[1] Veramed Ltd., Regal House, 70 London Road, Twickenham, UK
[2] Department of Biostatistics, GSK Research and Development, 1250 S Collegeville Road, Collegeville, Pennsylvania, USA
[3] Department of Biostatistics, GSK Research and Development, 79 New Oxford Street, London, UK

A Appendix

A.1 Code

A.1.1 Stan code in R

The following code can be used to fit the illustrative example from the main paper. The model below uses prior (iii), a multivariate mixture of two product distributions. Data is sampled with the MASS package (Venables and Ripley, 2002) and the model is fit with the rstan package (Stan Development Team, 2025) in R (R Core Team, 2025).

```
library(rstan)
library(MASS)

# Set Stan options
options(mc.cores = parallel::detectCores()) # uses all available cores
rstan_options(auto_write = TRUE)

# Generate the study data and set the hyperpriors
N <- 30                                    # simulate 30 patients
set.seed(10)
X <- mvrnorm(n = 30, c(6,8), matrix(c(18, 5, 5, 22),nrow=2),
           tol = 1e-6, empirical = TRUE)   # sample data
w_inf <- 0.5                               # weight on inf component
mu_inf <- c(5,5)                           # means of inf component
mu_vag <- c(5,5)                           # means of vague component
Sigma_inf <- matrix(c(1,0.5,0.5,1),nrow=2) # covariance of inf component
Sigma_vag <- matrix(c(20,10,10,20),nrow=2) # covariance of vag component
```

*Corresponding author: e-mail: benjamin.f.hartley@gsk.com

© 2010 WILEY-VCH Verlag GmbH & Co. KGaA, Weinheim

```
# Stan code
NtimesIWmodel <- "
functions {
  real mixture_multi_normal_cholesky_lpdf(vector param,
  real wt,
  vector mvect1, vector mvect2,
  matrix vmat1, matrix vmat2){
  return (
    log_sum_exp(
      log(wt) + multi_normal_cholesky_lpdf(param|mvect2,vmat1),
      log1m(wt) + multi_normal_cholesky_lpdf(param|mvect2,vmat2))
      );
  }
  }
data {
  int<lower=0> N;                // number of patients
  array[N] vector[2] X;          // N x 2 array of outcome vectors
  real w_inf;                    // weight on the informative component
  vector[2] mu_inf;              // means of informative component
  vector[2] mu_vag;              // means of vague component
  cov_matrix[2] Sigma_inf;       // covariance matrix of inf component
  cov_matrix[2] Sigma_vag;       // covariance matrix of vague component
  }
transformed data {
  matrix[2,2] L_Sigma_inf = cholesky_decompose(Sigma_inf);
  matrix[2,2] L_Sigma_vag = cholesky_decompose(Sigma_vag);
  matrix[2,2] L_Psi = cholesky_decompose([[20, 10], [10, 20]]);
  }
parameters {
  vector[2] mu;                     // mean parameters
  cholesky_factor_cov[2] L_Sigma;  // covariance parameters
  }
model {
  // Priors
  // Mixture prior on the mean parameters
  mu ~ mixture_multi_normal_cholesky(w_inf, mu_inf, mu_vag,
    L_Sigma_inf, L_Sigma_vag);
```

© 2010 WILEY-VCH Verlag GmbH & Co. KGaA, Weinheim

```
  // ``Mixture'' of the same IW distribution on the cov parameters
  L_Sigma ~ inv_wishart_cholesky(2, L_Psi);
  // Likelihood
  X ~ multi_normal_cholesky(mu, L_Sigma);
  }
generated quantities {
  matrix[2,2] Sigma = multiply_lower_tri_self_transpose(L_Sigma);
  }
  "

# Call the Stan model
fit <- stan(model_code = NtimesIWmodel,
  data=c("N","X","w_inf","mu_inf","mu_vag", "Sigma_inf","Sigma_vag"),
  seed = 1234, iter=1000000+2000, warmup=2000)

# Model results
print(fit)
```

Sampling models (i) and (ii) with Stan is also possible with modifications to the code. Additionally the model can be fit to partial study data by replacing `X ~ multi_normal_cholesky(mu, L_Sigma);` with `X[,1] ~ normal(mu[1], sqrt(Sigma[1,1]));`, (and moving the Sigma matrix from the generated quantities block to a transformed parameters block) for a one-dimensional likelihood update to a two-dimensional prior.

A.1.2 SAS code

The same posterior resulting from prior (iii) can be sampled using with the MCMC procedure in SAS, creating a dataset called `sample` with two columns `X1` and `X2` and running the following code.

```
proc mcmc data=sample outpost=outpost nbi=500 nmc=1000000 thin=10
seed=1234 propcov=quanew plots=none;
array X[2] X1 X2;
array mu[2] mu1 mu2;
array Sigma[2,2] Sigma11 Sigma12 Sigma21 Sigma22;
parms mu {5 5} Sigma {20 10 10 20} w_inf 1; *starting values;
begincnst;
  *informative component;
  array mu_inf[2] mu_inf1 mu_inf2 (5 5);
  rc = logmpdfsetsq('Sigma_inf', 1, 0.5, 0.5, 1);
  *vague component;
  array mu_vag[2] mu_vag1 mu_vag2 (5 5);
  rd = logmpdfsetsq('Sigma_vag', 20, 10, 10, 20);
```

© 2010 WILEY-VCH Verlag GmbH & Co. KGaA, Weinheim

```
  array Psi[2,2] (20 10 10 20); *prior scale matrix for IW;
endcnst;
beginprior;
  *equipoise prior weight;
  prior w_inf~binary(0.5);
  *mixture prior;
  lmvn =     w_inf*logmpdfnormal(of mu1-mu2,
                                 of mu_inf1-mu_inf2, 'Sigma_inf') +
        (1-w_inf)*logmpdfnormal(of mu1-mu2,
                                 of mu_vag1-mu_vag2, 'Sigma_vag');
  prior mu ~ general(lmvn);
  *residual variance prior;
  prior Sigma ~ iwishart(2, Psi); *prior of nu=2 for both components;
endprior;
model X ~ mvn(mu, Sigma);
run;
```

Setting the `V2` variable to missing in a prior datastep fits the partial study data model. Sampling models (i) and (ii) with the MCMC procedure is also possible with modifications to the code. However, mixing a normal-inverse-Wishart prior to fit model (ii) in a single analysis appears complicated because the logmpdfsetsq function used to construct prior matrices does not accept model parameters as arguments. However, one can run two fixed prior models and combine the posteriors "by hand" using the correct marginal likelihood posterior weight.

A.2 Theorems and proofs

The marginal likelihood of the data $p(\boldsymbol{x})$ is the normalising constant in Bayes' theorem. It is sometimes called the model evidence because it describes the probability of the data model. The marginal likelihood can be found by integrating the likelihood function of the current study data $\boldsymbol{x}_i \in \mathbb{R}^J,\ i \in \{1, 2, \ldots, n\}$ over the density of the prior on the mean parameter vector on the full support of the domain for the mean. Posterior weights on the components of a mixture prior are proportional to the prior weights multiplied by the marginal likelihood or model evidence for the prior component (Bernardo and Smith, 2000).

A.2.1 Marginal likelihood and posterior weights for an MVN prior and MVN likelihood function

Theorem A.1 *Let $\boldsymbol{\mu}$, a vector of $J+K$ mean parameters be partitioned into two vectors, $\boldsymbol{\alpha}$ of length J and $\boldsymbol{\beta}$ of length K. Define a multivariate normal prior distribution on $\boldsymbol{\mu}$ such that $p(\boldsymbol{\mu}) = \varphi(\boldsymbol{\mu}; \boldsymbol{\mu_0}, \boldsymbol{\Sigma_0}) = \varphi(\left(\begin{smallmatrix}\boldsymbol{\alpha}\\ \boldsymbol{\beta}\end{smallmatrix}\right); \left(\begin{smallmatrix}\boldsymbol{\alpha_0}\\ \boldsymbol{\beta_0}\end{smallmatrix}\right), \left(\begin{smallmatrix}\mathbf{A_0} & \mathbf{B_0}\\ \mathbf{C_0} & \mathbf{D_0}\end{smallmatrix}\right))$. Let $\boldsymbol{x}_i$ for $i = 1, 2, ..., n$ be i.i.d. outcome vectors observed on the first J dimensions. Let the variance of the $\boldsymbol{x}_i$ be known and equal to $\mathbf{A}$, and denote the mean of the $\boldsymbol{x}_i$ by $\bar{\boldsymbol{x}}$. Then for a multivariate normal likelihood function, the marginal likelihood $p(\boldsymbol{x})$ is given by*

$$p(\boldsymbol{x}) = (2\pi)^{\frac{J}{2}} \left|\frac{1}{n}\mathbf{A}\right|^{\frac{1}{2}} \left[\prod_{1}^{n} \varphi(\boldsymbol{x}_i; \bar{\boldsymbol{x}}, \mathbf{A})\right] \varphi\left(\bar{\boldsymbol{x}}; \boldsymbol{\alpha_0}, \mathbf{A_0} + \frac{1}{n}\mathbf{A}\right) \tag{1}$$

© 2010 WILEY-VCH Verlag GmbH & Co. KGaA, Weinheim

P r o o f. Denote the domain of the mean parameter $\mathcal{M} = \mathbb{R}^{J+K}$. The marginal likelihood is defined as follows

$$\begin{aligned} p(\boldsymbol{x}) &= \int_{\mathcal{M}} p(\boldsymbol{x}, \boldsymbol{\mu})\, d\boldsymbol{\mu} \\ &= \int_{\mathcal{M}} p(\boldsymbol{x}|\boldsymbol{\alpha})\; p(\boldsymbol{\mu})\, d\boldsymbol{\mu} \\ &= \int_{\mathcal{M}} \left[\prod_{1}^{n} \varphi(\boldsymbol{x}_i; \boldsymbol{\alpha}, \mathbf{A})\right] \varphi(\boldsymbol{\mu}; \boldsymbol{\mu_0}, \boldsymbol{\Sigma_0})\, d\boldsymbol{\mu}. \end{aligned}$$

The integral can be rewritten by noting that for appropriately dimensioned vectors $\mathbf{v_1}$ and $\mathbf{v_2}$ and a matrix $\mathbf{S_1}$, the identity $(\mathbf{v_1} - \mathbf{v_2})^{\mathsf{T}}\mathbf{S_1}(\mathbf{v_1} - \mathbf{v_2}) \equiv (\mathbf{v_2} - \mathbf{v_1})^{\mathsf{T}}\mathbf{S_1}(\mathbf{v_2} - \mathbf{v_1})$, implies $\varphi(\mathbf{v_1}; \mathbf{v_2}, \mathbf{S_1}) \equiv \varphi(\mathbf{v_2}; \mathbf{v_1}, \mathbf{S_1})$. Additionally, denote $\mathbf{S_0} = \mathbf{D_0} - \mathbf{C_0}\mathbf{A_0}^{-1}\mathbf{B_0}$ the Schur complement of $\mathbf{A_0}$ in $\boldsymbol{\Sigma_0}$, and $p(\boldsymbol{\mu})$ can be factorised into $p(\boldsymbol{\alpha})p(\boldsymbol{\beta}|\boldsymbol{\alpha})$ as follows

$$p(\boldsymbol{x}) = \int_{\mathcal{M}} \left[\prod_{1}^{n} \varphi(\boldsymbol{\alpha}; \boldsymbol{x}_i, \mathbf{A})\right] \varphi(\boldsymbol{\alpha}; \boldsymbol{\alpha_0}, \mathbf{A_0})\, \varphi\big(\boldsymbol{\beta}; \boldsymbol{\beta_0} - \mathbf{C_0}\mathbf{A_0}^{-1}(\boldsymbol{\alpha} - \boldsymbol{\alpha_0}), \mathbf{S_0}\big)\, d\boldsymbol{\mu}.$$

The product of MVN density functions can be rewritten with the following identity, where $\mathbf{v_1}$, $\mathbf{v_2}$ and $\mathbf{v_3}$ are any vectors, and $\mathbf{S_1}$ and $\mathbf{S_2}$ are any (symmetric) matrices of the same dimension,

$$\begin{aligned} &\varphi(\mathbf{v_3}; \mathbf{v_1}, \mathbf{S_1})\, \varphi(\mathbf{v_3}; \mathbf{v_2}, \mathbf{S_2}) \\ &\qquad \equiv \varphi(\mathbf{v_2}; \mathbf{v_1}, \mathbf{S_1} + \mathbf{S_2})\, \varphi\Big(\mathbf{v_3}; \big(\mathbf{S_1}^{-1} + \mathbf{S_2}^{-1}\big)^{-1}\big(\mathbf{S_1}^{-1}\mathbf{v_1} + \mathbf{S_2}^{-1}\mathbf{v_2}\big), \big(\mathbf{S_1}^{-1} + \mathbf{S_2}^{-1}\big)^{-1}\Big). \end{aligned}$$

Define $\bar{\boldsymbol{x}}_m$ as the average of the first m vectors of the observed data $\bar{\boldsymbol{x}}_m = \frac{1}{m}\sum_{i=1}^{m} \boldsymbol{x}_i$, and using the above identity n times it can be shown that

$$\begin{aligned} \left[\prod_{1}^{n} \varphi(\boldsymbol{\alpha}; \boldsymbol{x}_i, \mathbf{A})\right] \varphi(\boldsymbol{\alpha}; \boldsymbol{\alpha_0}, \mathbf{A_0}) &= \left[\prod_{2}^{n} \varphi\Big(\boldsymbol{x}_i; \bar{\boldsymbol{x}}_{i-1}, \frac{i}{i-1}\mathbf{A}\Big)\right] \varphi\Big(\bar{\boldsymbol{x}}; \boldsymbol{\alpha_0}, \mathbf{A_0} + \frac{1}{n}\mathbf{A}\Big) \\ &\quad \varphi\Big(\boldsymbol{\alpha}; \big(\mathbf{A_0}^{-1} + n\mathbf{A}^{-1}\big)^{-1}\big(\mathbf{A_0}^{-1}\boldsymbol{\alpha_0} + n\mathbf{A}^{-1}\bar{\boldsymbol{x}}\big), \big(\mathbf{A_0}^{-1} + n\mathbf{A}^{-1}\big)^{-1}\Big) \end{aligned}$$

The first bracketed term on the left hand side of is a function of the data $\boldsymbol{x}$ and the (known) covariance matrix $\mathbf{A}$ and can be rewritten (by induction) using another identity, $\mathbf{v_1}^{\mathsf{T}}\mathbf{S_1}\mathbf{v_1} \equiv \mathrm{tr}(\mathbf{S_1}\mathbf{v_1}\mathbf{v_1}^{\mathsf{T}})$, as follows

$$\prod_{2}^{n} \varphi\Big(\boldsymbol{x}_i; \bar{\boldsymbol{x}}_{i-1}, \frac{i}{i-1}\mathbf{A}\Big) = (2\pi)^{\frac{J}{2}} \left|\frac{1}{n}\mathbf{A}\right|^{\frac{1}{2}} \prod_{1}^{n} \varphi(\boldsymbol{x}_i; \bar{\boldsymbol{x}}, \mathbf{A}).$$

Substituting this into the expression for the marginal likelihood gives

$$\begin{aligned} p(\boldsymbol{x}) = \int_{\mathcal{M}} &(2\pi)^{\frac{J}{2}} \left|\frac{1}{n}\mathbf{A}\right|^{\frac{1}{2}} \left[\prod_{1}^{n} \varphi(\boldsymbol{x}_i; \bar{\boldsymbol{x}}, \mathbf{A})\right] \varphi\Big(\bar{\boldsymbol{x}}; \boldsymbol{\alpha_0}, \mathbf{A_0} + \frac{1}{n}\mathbf{A}\Big) \\ &\varphi\Big(\boldsymbol{\alpha}; \big(\mathbf{A_0}^{-1} + n\mathbf{A}^{-1}\big)^{-1}\big(\mathbf{A_0}^{-1}\boldsymbol{\alpha_0} + n\mathbf{A}^{-1}\bar{\boldsymbol{x}}\big), \big(\mathbf{A_0}^{-1} + n\mathbf{A}^{-1}\big)^{-1}\Big) \\ &\varphi\big(\boldsymbol{\beta}; \boldsymbol{\beta_0} - \mathbf{C_0}\mathbf{A_0}^{-1}(\boldsymbol{\alpha} - \boldsymbol{\alpha_0}), \mathbf{S_0}\big)\, d\boldsymbol{\mu}. \end{aligned}$$

The first line depends only on the data $\boldsymbol{x}$, and the covariance matrix $\mathbf{A}$ and so can be passed outside the integral. Remaining inside the integral is a (factorised) MVN distribution $p(\boldsymbol{\mu}) = p(\boldsymbol{\alpha})p(\boldsymbol{\beta}|\boldsymbol{\alpha})$ which

© 2010 WILEY-VCH Verlag GmbH & Co. KGaA, Weinheim

integrates to 1 leaving the marginal likelihood given in (1) as follows

$$
\begin{aligned}
p(\boldsymbol{x}) =& (2\pi)^{\frac{J}{2}} \left| \frac{1}{n}\mathbf{A} \right|^{\frac{1}{2}} \left[\prod_{1}^{n} \varphi(\boldsymbol{x}_i; \bar{\boldsymbol{x}}, \mathbf{A}) \right] \varphi\Big(\bar{\boldsymbol{x}}; \boldsymbol{\alpha_0}, \mathbf{A_0} + \frac{1}{n}\mathbf{A} \Big) \\
& \int_{\mathcal{M}} \varphi\Big(\boldsymbol{\alpha}; \big(\mathbf{A_0}^{-1} + n\mathbf{A}^{-1}\big)^{-1} \big(\mathbf{A_0}^{-1}\boldsymbol{\alpha_0} + n\mathbf{A}^{-1}\bar{\boldsymbol{x}}\big), \big(\mathbf{A_0}^{-1} + n\mathbf{A}^{-1}\big)^{-1} \Big) \\
& \varphi\big(\boldsymbol{\beta}; \boldsymbol{\beta_0} - \mathbf{C_0}\mathbf{A_0}^{-1}(\boldsymbol{\alpha} - \boldsymbol{\alpha_0}), \mathbf{S_0} \big) \, d\boldsymbol{\mu} \\
=& (2\pi)^{\frac{J}{2}} \left| \frac{1}{n}\mathbf{A} \right|^{\frac{1}{2}} \left[\prod_{1}^{n} \varphi(\boldsymbol{x}_i; \bar{\boldsymbol{x}}, \mathbf{A}) \right] \varphi\Big(\bar{\boldsymbol{x}}; \boldsymbol{\alpha_0}, \mathbf{A_0} + \frac{1}{n}\mathbf{A} \Big).
\end{aligned}
$$

□

Corollary A.2 *Using the same assumptions as Theorem A.1 and analogous subvector and submatrix notation for two sets of hyperparameters, the posterior weight from a two component multivariate normal mixture prior on the mean parameter* $p(\boldsymbol{\mu}) = \sum_c w_c \, \varphi(\boldsymbol{\mu}; \boldsymbol{\mu}_c, \boldsymbol{\Sigma}_c)$ *with* $c \in \{inf, vag\}$ *is given by (2),*

$$
w'_{\text{inf}} = \frac{w_{\text{inf}} \, \varphi\big(\bar{\boldsymbol{x}}; \boldsymbol{\alpha}_{\textbf{inf}}, \mathbf{A}_{\textbf{inf}} + \frac{1}{n}\mathbf{A}\big)}{w_{\text{inf}} \, \varphi\big(\bar{\boldsymbol{x}}; \boldsymbol{\alpha}_{\textbf{inf}}, \mathbf{A}_{\textbf{inf}} + \frac{1}{n}\mathbf{A}\big) + w_{\text{vag}} \, \varphi\big(\bar{\boldsymbol{x}}; \boldsymbol{\alpha}_{\textbf{vag}}, \mathbf{A}_{\textbf{vag}} + \frac{1}{n}\mathbf{A}\big)}. \tag{2}
$$

The result follows from posterior weights being proportional to the marginal likelihood (or model evidence) found in Theorem A.1 multiplied by the prior weight (Bernardo and Smith, 2000). Using (1) posterior weights for an informative and a vague distribution component can be written

$$
\begin{aligned}
w'_{\text{inf}} &\propto w_{\text{inf}} (2\pi)^{\frac{J}{2}} \left| \frac{1}{n}\mathbf{A} \right|^{\frac{1}{2}} \left[\prod_{1}^{n} \varphi(\boldsymbol{x}_i; \bar{\boldsymbol{x}}, \mathbf{A}) \right] \varphi\Big(\bar{\boldsymbol{x}}; \boldsymbol{\alpha}_{\textbf{inf}}, \mathbf{A}_{\textbf{inf}} + \frac{1}{n}\mathbf{A} \Big), \\
w'_{\text{vag}} &\propto w_{\text{vag}} (2\pi)^{\frac{J}{2}} \left| \frac{1}{n}\mathbf{A} \right|^{\frac{1}{2}} \left[\prod_{1}^{n} \varphi(\boldsymbol{x}_i; \bar{\boldsymbol{x}}, \mathbf{A}) \right] \varphi\Big(\bar{\boldsymbol{x}}; \boldsymbol{\alpha}_{\textbf{vag}}, \mathbf{A}_{\textbf{vag}} + \frac{1}{n}\mathbf{A} \Big).
\end{aligned}
$$

The expression $(2\pi)^{\frac{J}{2}} |\frac{1}{n}\mathbf{A}|^{\frac{1}{2}} [\prod_1^n \varphi(\boldsymbol{x}_i; \bar{\boldsymbol{x}}, \mathbf{A})]$ depends only on the observed data and the known covariance matrix and is a common factor in both weights, this cancels giving (2). Additionally, Using a plug-in estimator $\hat{\boldsymbol{\Sigma}}$ for the covariance matrix $\mathbf{A}$ gives the following expression

$$
\hat{w}'_{\text{inf}} = \frac{w_{\text{inf}} \, \varphi\Big(\bar{\boldsymbol{x}}; \boldsymbol{\alpha}_{\textbf{inf}}, \mathbf{A}_{\textbf{inf}} + \frac{1}{n}\hat{\boldsymbol{\Sigma}}\Big)}{w_{\text{inf}} \, \varphi\Big(\bar{\boldsymbol{x}}; \boldsymbol{\alpha}_{\textbf{inf}}, \mathbf{A}_{\textbf{inf}} + \frac{1}{n}\hat{\boldsymbol{\Sigma}}\Big) + w_{\text{vag}} \, \varphi\Big(\bar{\boldsymbol{x}}; \boldsymbol{\alpha}_{\textbf{vag}}, \mathbf{A}_{\textbf{vag}} + \frac{1}{n}\hat{\boldsymbol{\Sigma}}\Big)}.
$$

In the $K = 0$ case, when all visits are observed, this becomes

$$
\hat{w}'_{\text{inf}} = \frac{w_{\text{inf}} \, \varphi\Big(\bar{\boldsymbol{x}}; \boldsymbol{\mu}_{\textbf{inf}}, \boldsymbol{\Sigma}_{\textbf{inf}} + \frac{1}{n}\hat{\boldsymbol{\Sigma}}\Big)}{w_{\text{inf}} \, \varphi\Big(\bar{\boldsymbol{x}}; \boldsymbol{\mu}_{\textbf{inf}}, \boldsymbol{\Sigma}_{\textbf{inf}} + \frac{1}{n}\hat{\boldsymbol{\Sigma}}\Big) + w_{\text{vag}} \, \varphi\Big(\bar{\boldsymbol{x}}; \boldsymbol{\mu}_{\textbf{vag}}, \boldsymbol{\Sigma}_{\textbf{vag}} + \frac{1}{n}\hat{\boldsymbol{\Sigma}}\Big)}.
$$

A.2.2 Marginal likelihood and posterior weights for an NIW prior and MVN likelihood function

Theorem A.3 *Let* $\boldsymbol{\mu}$*, a vector of* $J + K$ *mean parameters be partitioned into two vectors,* $\boldsymbol{\alpha}$ *of length* J *and* $\boldsymbol{\beta}$ *of length* K*. Let* $\boldsymbol{\Sigma}$ *the matrix of covariance parameters be conformably partitioned*

© 2010 WILEY-VCH Verlag GmbH & Co. KGaA, Weinheim

into blocks, $\boldsymbol{\Sigma} = \left(\begin{smallmatrix}\mathbf{A} & \mathbf{B}\\ \mathbf{C} & \mathbf{D}\end{smallmatrix}\right)$*. Define a multivariate normal-inverse-Wishart prior distribution on* $(\boldsymbol{\mu}, \boldsymbol{\Sigma})$ *such that* $p(\boldsymbol{\mu}, \boldsymbol{\Sigma}) = \varphi(\boldsymbol{\mu}; \boldsymbol{\mu_0}, \boldsymbol{\Sigma}/\lambda_0)\,\omega(\boldsymbol{\Sigma}; \boldsymbol{\Psi_0}, \nu_0) = \varphi(\left(\begin{smallmatrix}\boldsymbol{\alpha}\\ \boldsymbol{\beta}\end{smallmatrix}\right); \left(\begin{smallmatrix}\boldsymbol{\alpha_0}\\ \boldsymbol{\beta_0}\end{smallmatrix}\right), \left(\begin{smallmatrix}\mathbf{A} & \mathbf{B}\\ \mathbf{C} & \mathbf{D}\end{smallmatrix}\right)/\lambda_0)\,\omega(\left(\begin{smallmatrix}\mathbf{A} & \mathbf{B}\\ \mathbf{C} & \mathbf{D}\end{smallmatrix}\right); \left(\begin{smallmatrix}\mathbf{A_0} & \mathbf{B_0}\\ \mathbf{C_0} & \mathbf{D_0}\end{smallmatrix}\right), \nu_0)$*. Let* $\boldsymbol{x}_i$ *for* $i = 1, 2, ..., n$ *be i.i.d. outcome vectors observed on the first* J *dimensions. Denote the mean of the* $\boldsymbol{x}_i$ *by* $\bar{\boldsymbol{x}}$*. Then for a multivariate normal likelihood function, the marginal likelihood* $p(\boldsymbol{x})$ *is given by*

$$p(\boldsymbol{x}) = \pi^{-\frac{nJ}{2}} \left(\frac{\lambda_0'}{\lambda_0}\right)^{-\frac{J}{2}} \frac{\Gamma_J\left(\frac{\nu_0'-K}{2}\right) |\mathbf{A_0}|^{\frac{\nu_0-K}{2}}}{\Gamma_J\left(\frac{\nu_0-K}{2}\right) |\mathbf{A_0'}|^{\frac{\nu_0'-K}{2}}} \tag{3}$$

where $\mathbf{A_0'} = \mathbf{A_0} + \left[\sum_1^n (\boldsymbol{x}_i - \bar{\boldsymbol{x}})(\boldsymbol{x}_i - \bar{\boldsymbol{x}})^{\mathsf{T}}\right] + \dfrac{\lambda_0 n}{\lambda_0 + n}(\boldsymbol{\alpha_0} - \bar{\boldsymbol{x}})(\boldsymbol{\alpha_0} - \bar{\boldsymbol{x}})^{\mathsf{T}}$*,* $\lambda_0' = \lambda_0 + n$ *and* $\nu_0' = \nu_0 + n$*.*

Proof. Denote the domain of the mean parameter vector by $\mathcal{M} = \mathbb{R}^{J+K}$, and the domain of the covariance matrix by $\mathcal{S} \subset \mathbb{R}^{(J+K)\times(J+K)}$ (such that the covariance matrix is positive definite). The marginal likelihood can be written as follows

$$\begin{aligned}
p(\boldsymbol{x}) &= \iint_{\mathcal{S}\times\mathcal{M}} p(\boldsymbol{x}, \boldsymbol{\mu}, \boldsymbol{\Sigma})\, d\boldsymbol{\mu}\, d\boldsymbol{\Sigma} \\
&= \iint_{\mathcal{S}\times\mathcal{M}} p(\boldsymbol{x}|\boldsymbol{\alpha}, \mathbf{A})\; p(\boldsymbol{\mu}, \boldsymbol{\Sigma})\, d\boldsymbol{\mu}\, d\boldsymbol{\Sigma} \\
&= \iint_{\mathcal{S}\times\mathcal{M}} \left[\prod_1^n \varphi(\boldsymbol{x}_i; \boldsymbol{\alpha}, \mathbf{A})\right] \varphi\left(\boldsymbol{\mu}; \boldsymbol{\mu_0}, \frac{\boldsymbol{\Sigma}}{\lambda_0}\right) \omega(\boldsymbol{\Sigma}; \boldsymbol{\Psi_0}, \nu_0)\, d\boldsymbol{\mu}\, d\boldsymbol{\Sigma} \\
&= \iint_{\mathcal{S}\times\mathcal{M}} \left[\prod_1^n \varphi(\boldsymbol{\alpha}; \boldsymbol{x}_i, \mathbf{A})\right] \varphi\left(\boldsymbol{\alpha}; \boldsymbol{\alpha_0}, \frac{\mathbf{A}}{\lambda_0}\right) p(\boldsymbol{\beta}|\boldsymbol{\alpha})\, \omega(\boldsymbol{\Sigma}; \boldsymbol{\Psi_0}, \nu_0)\, d\boldsymbol{\mu}\, d\boldsymbol{\Sigma}
\end{aligned}$$

The conditional distribution $p(\boldsymbol{\beta}|\boldsymbol{\alpha})$ is known to be MVN (see Appendix A.2.1) but the form is not important in this context since it will be integrated out anyway. The marginal distribution of $\boldsymbol{\alpha}$ is MVN with mean $\boldsymbol{\alpha_0}$ and variance $\mathbf{A}/\lambda_0$ and the product of MVN density functions can be rewritten as before

$$\begin{aligned}
&\left[\prod_1^n \varphi(\boldsymbol{\alpha}; \boldsymbol{x}_i, \mathbf{A})\right] \varphi\left(\boldsymbol{\alpha}; \boldsymbol{\alpha_0}, \frac{\mathbf{A}}{\lambda_0}\right) \\
&\qquad = (2\pi)^{\frac{J}{2}} \left|\frac{1}{n}\mathbf{A}\right|^{\frac{1}{2}} \prod_1^n \varphi(\boldsymbol{x}_i; \bar{\boldsymbol{x}}, \mathbf{A})\, \varphi\left(\bar{\boldsymbol{x}}; \boldsymbol{\alpha_0}, \frac{\lambda_0 + n}{\lambda_0 n}\mathbf{A}\right) \varphi\left(\boldsymbol{\alpha}; \frac{\lambda_0\boldsymbol{\alpha_0} + n\bar{\boldsymbol{x}}}{\lambda_0 + n}, \frac{\mathbf{A}}{\lambda_0 + n}\right)
\end{aligned}$$

Substituting this into the expression for the marginal likelihood and noting that the factorised MVN distribution $p(\boldsymbol{\mu}) = p(\boldsymbol{\alpha})p(\boldsymbol{\beta}|\boldsymbol{\alpha})$ will integrate to 1 and that no other terms depend on the mean parameters gives

$$\begin{aligned}
p(\boldsymbol{x}) &= \iint_{\mathcal{S}\times\mathcal{M}} (2\pi)^{\frac{J}{2}} \left|\frac{1}{n}\mathbf{A}\right|^{\frac{1}{2}} \prod_1^n \varphi(\boldsymbol{x}_i; \bar{\boldsymbol{x}}, \mathbf{A})\, \varphi\left(\bar{\boldsymbol{x}}; \boldsymbol{\alpha_0}, \frac{\lambda_0 + n}{\lambda_0 n}\mathbf{A}\right) \\
&\qquad \varphi\left(\boldsymbol{\alpha}; \frac{\lambda_0\boldsymbol{\alpha_0} + n\bar{\boldsymbol{x}}}{\lambda_0 + n}, \frac{\mathbf{A}}{\lambda_0 + n}\right) p(\boldsymbol{\beta}|\boldsymbol{\alpha})\, \omega(\boldsymbol{\Sigma}; \boldsymbol{\Psi_0}, \nu_0)\, d\boldsymbol{\mu}\, d\boldsymbol{\Sigma} \\
&= \int_{\mathcal{S}} (2\pi)^{\frac{J}{2}} \left|\frac{1}{n}\mathbf{A}\right|^{\frac{1}{2}} \left[\prod_1^n \varphi(\boldsymbol{x}_i; \bar{\boldsymbol{x}}, \mathbf{A})\right] \varphi\left(\bar{\boldsymbol{x}}; \boldsymbol{\alpha_0}, \frac{\lambda_0 + n}{\lambda_0 n}\mathbf{A}\right) \omega(\boldsymbol{\Sigma}; \boldsymbol{\Psi_0}, \nu_0)\, d\boldsymbol{\Sigma}
\end{aligned}$$

Similarly, factorise the inverse-Wishart density function for $\boldsymbol{\Sigma}$ into the marginal density for $\mathbf{A}$ which is known to also be inverse-Wishart with a conformably reduced scale matrix but with a degree of freedom

© 2010 WILEY-VCH Verlag GmbH & Co. KGaA, Weinheim

parameter reduced by K, *i.e.* the number of dimensions being removed (Gupta and Nagar, 2018), and a conditional density function whose form is not important because it will be integrated out $\omega(\mathbf{\Sigma};\mathbf{\Psi_0},\nu_0) = \omega(\mathbf{A};\mathbf{A_0},\nu_0-K)\,p(\mathbf{BCD}|\mathbf{A})$ as follows

$$
\begin{aligned}
p(\boldsymbol{x}) = & \int_{\mathcal{S}} (2\pi)^{\frac{J}{2}} \left|\frac{1}{n}\mathbf{A}\right|^{\frac{1}{2}} \left[\prod_{1}^{n} \varphi(\boldsymbol{x}_i;\bar{\boldsymbol{x}},\mathbf{A})\right] \varphi\left(\bar{\boldsymbol{x}};\boldsymbol{\alpha_0},\frac{\lambda_0+n}{\lambda_0 n}\mathbf{A}\right) \\
& \omega(\mathbf{A};\mathbf{A_0},\nu_0-K)\,p(\mathbf{BCD}|\mathbf{A})\,d\mathbf{\Sigma} \\
= & \int_{\mathcal{S}} (2\pi)^{\frac{J}{2}}(2\pi)^{-\frac{nJ}{2}}(2\pi)^{-\frac{J}{2}} \left|\frac{1}{n}\mathbf{A}\right|^{\frac{1}{2}} |\mathbf{A}|^{-\frac{n}{2}} \left|\frac{\lambda_0+n}{\lambda_0 n}\mathbf{A}\right|^{-\frac{1}{2}} \frac{|\mathbf{A_0}|^{\frac{\nu_0-K}{2}}|\mathbf{A}|^{-\frac{\nu_0-K+J+1}{2}}}{2^{\frac{(\nu_0-K)J}{2}}\Gamma_J\left(\frac{\nu_0-K}{2}\right)} \\
& \left[\prod_{1}^{n} \exp\left(-\frac{1}{2}(\boldsymbol{x}_i-\bar{\boldsymbol{x}})^{\mathsf{T}}\mathbf{A}^{-1}(\boldsymbol{x}_i-\bar{\boldsymbol{x}})\right)\right] \exp\left(-\frac{1}{2}\cdot\frac{\lambda_0 n}{\lambda_0+n}(\boldsymbol{\alpha_0}-\bar{\boldsymbol{x}})^{\mathsf{T}}\mathbf{A}^{-1}(\boldsymbol{\alpha_0}-\bar{\boldsymbol{x}})\right) \\
& \exp\left(-\frac{1}{2}\operatorname{tr}\left(\mathbf{A_0}\mathbf{A}^{-1}\right)\right) p(\mathbf{BCD}|\mathbf{A})\,d\mathbf{\Sigma} \\
= & \int_{\mathcal{S}} (2\pi)^{-\frac{nJ}{2}} \left(\frac{\lambda_0+n}{\lambda_0}\right)^{-\frac{J}{2}} \frac{|\mathbf{A_0}|^{\frac{\nu_0-K}{2}}|\mathbf{A}|^{-\frac{\nu_0-K+n+J+1}{2}}}{2^{\frac{(\nu_0-K)J}{2}}\Gamma_J\left(\frac{\nu_0-K}{2}\right)} \\
& \exp\left(-\frac{1}{2}\operatorname{tr}\left(\mathbf{A}^{-1}\left(\left[\sum_{1}^{n}(\boldsymbol{x}_i-\bar{\boldsymbol{x}})(\boldsymbol{x}_i-\bar{\boldsymbol{x}})^{\mathsf{T}}\right] + \frac{\lambda_0 n}{\lambda_0+n}(\boldsymbol{\alpha_0}-\bar{\boldsymbol{x}})(\boldsymbol{\alpha_0}-\bar{\boldsymbol{x}})^{\mathsf{T}} + \mathbf{A_0}\right)\right)\right) \\
& p(\mathbf{BCD}|\mathbf{A})\,d\mathbf{\Sigma}
\end{aligned}
$$

This expression can be rewritten using the definitions given above for the updated parameters and by integrating the inverse-Wishart density function to 1 leaving the marginal likelihood given by (3),

$$
\begin{aligned}
p(\boldsymbol{x}) = & \int_{\mathcal{S}} \pi^{-\frac{nJ}{2}} \left(\frac{\lambda_0'}{\lambda_0}\right)^{-\frac{J}{2}} \frac{|\mathbf{A_0}|^{\frac{\nu_0-K}{2}}|\mathbf{A}|^{-\frac{\nu_0'-K+J+1}{2}}}{2^{\frac{(\nu_0'-K)J}{2}}\Gamma_J\left(\frac{\nu_0-K}{2}\right)} \exp\left(-\frac{1}{2}\operatorname{tr}\left(\mathbf{A}^{-1}\mathbf{A_0'}\right)\right) p(\mathbf{BCD}|\mathbf{A})\,d\mathbf{\Sigma} \\
= & \pi^{-\frac{nJ}{2}} \left(\frac{\lambda_0'}{\lambda_0}\right)^{-\frac{J}{2}} \frac{\Gamma_J\left(\frac{\nu_0'-K}{2}\right)|\mathbf{A_0}|^{\frac{\nu_0-K}{2}}}{\Gamma_J\left(\frac{\nu_0-K}{2}\right)|\mathbf{A_0'}|^{\frac{\nu_0'-K}{2}}} \int_{\mathcal{S}} \omega\left(\mathbf{A};\mathbf{A_0'},\nu_0'-K\right) p(\mathbf{BCD}|\mathbf{A})\,d\mathbf{\Sigma} \\
= & \pi^{-\frac{nJ}{2}} \left(\frac{\lambda_0'}{\lambda_0}\right)^{-\frac{J}{2}} \frac{\Gamma_J\left(\frac{\nu_0'-K}{2}\right)|\mathbf{A_0}|^{\frac{\nu_0-K}{2}}}{\Gamma_J\left(\frac{\nu_0-K}{2}\right)|\mathbf{A_0'}|^{\frac{\nu_0'-K}{2}}}
\end{aligned}
$$

□

Corollary A.4 *Using the same assumptions as Theorem A.3 and analogous subvector and submatrix notation for two sets of hyperparameters, the posterior weight from a two component mixture prior* $p(\boldsymbol{\mu},\mathbf{\Sigma}) = \sum_c w_c\,\varphi(\boldsymbol{\mu};\boldsymbol{\mu}_c,\mathbf{\Sigma}/\lambda_c)\,\omega(\mathbf{\Sigma};\mathbf{\Psi}_c,\nu_c)$ *with* $c \in \{\textit{inf},\textit{vag}\}$ *is given by (4),*

© 2010 WILEY-VCH Verlag GmbH & Co. KGaA, Weinheim

$$w'_{\text{inf}} = \frac{w_{\text{inf}}\left(\frac{\lambda'_{\text{inf}}}{\lambda_{\text{inf}}}\right)^{-\frac{J}{2}} \frac{\Gamma_J\left(\frac{\nu'_{\text{inf}}-K}{2}\right)|\mathbf{A_{inf}}|^{\frac{\nu_{\text{inf}}-K}{2}}}{\Gamma_J\left(\frac{\nu_{\text{inf}}-K}{2}\right)|\mathbf{A'_{inf}}|^{\frac{\nu'_{\text{inf}}-K}{2}}}}{w_{\text{inf}}\left(\frac{\lambda'_{\text{inf}}}{\lambda_{\text{inf}}}\right)^{-\frac{J}{2}} \frac{\Gamma_J\left(\frac{\nu'_{\text{inf}}-K}{2}\right)|\mathbf{A_{inf}}|^{\frac{\nu_{\text{inf}}-K}{2}}}{\Gamma_J\left(\frac{\nu_{\text{inf}}-K}{2}\right)|\mathbf{A'_{inf}}|^{\frac{\nu'_{\text{inf}}-K}{2}}} + w_{\text{vag}}\left(\frac{\lambda'_{\text{vag}}}{\lambda_{\text{vag}}}\right)^{-\frac{J}{2}} \frac{\Gamma_J\left(\frac{\nu'_{\text{vag}}-K}{2}\right)|\mathbf{A_{vag}}|^{\frac{\nu_{\text{vag}}-K}{2}}}{\Gamma_J\left(\frac{\nu_{\text{vag}}-K}{2}\right)|\mathbf{A'_{vag}}|^{\frac{\nu'_{\text{vag}}-K}{2}}}}. \tag{4}$$

As before, the result follows from posterior weights being proportional to the marginal likelihood (or model evidence) found in Theorem A.3 multiplied by the prior weight (Bernardo and Smith, 2000). Using (3) posterior weights for an informative and a vague distribution component can be written

$$w'_{\text{inf}} \propto w_{\text{inf}}\pi^{-\frac{nJ}{2}}\left(\frac{\lambda'_{\text{inf}}}{\lambda_{\text{inf}}}\right)^{-\frac{J}{2}} \frac{\Gamma_J\left(\frac{\nu'_{\text{inf}}-K}{2}\right)|\mathbf{A_{inf}}|^{\frac{\nu_{\text{inf}}-K}{2}}}{\Gamma_J\left(\frac{\nu_{\text{inf}}-K}{2}\right)|\mathbf{A'_{inf}}|^{\frac{\nu'_{\text{inf}}-K}{2}}}$$

$$w'_{\text{vag}} \propto w_{\text{vag}}\pi^{-\frac{nJ}{2}}\left(\frac{\lambda'_{\text{vag}}}{\lambda_{\text{vag}}}\right)^{-\frac{J}{2}} \frac{\Gamma_J\left(\frac{\nu'_{\text{vag}}-K}{2}\right)|\mathbf{A_{vag}}|^{\frac{\nu_{\text{vag}}-K}{2}}}{\Gamma_J\left(\frac{\nu_{\text{vag}}-K}{2}\right)|\mathbf{A'_{vag}}|^{\frac{\nu'_{\text{vag}}-K}{2}}}$$

The $\pi^{-nJ/2}$ terms cancel, giving the expression for the posterior weight in (4). Additionally when $K = 0$, *i.e.* all visits are observed, the posterior weight becomes

$$w'_{\text{inf}} = \frac{w_{\text{inf}}\left(\frac{\lambda'_{\text{inf}}}{\lambda_{\text{inf}}}\right)^{-\frac{J}{2}} \frac{\Gamma_J\left(\frac{\nu'_{\text{inf}}}{2}\right)|\mathbf{\Psi_{inf}}|^{\frac{\nu_{\text{inf}}}{2}}}{\Gamma_J\left(\frac{\nu_{\text{inf}}}{2}\right)|\mathbf{\Psi'_{inf}}|^{\frac{\nu'_{\text{inf}}}{2}}}}{w_{\text{inf}}\left(\frac{\lambda'_{\text{inf}}}{\lambda_{\text{inf}}}\right)^{-\frac{J}{2}} \frac{\Gamma_J\left(\frac{\nu'_{\text{inf}}}{2}\right)|\mathbf{\Psi_{inf}}|^{\frac{\nu_{\text{inf}}}{2}}}{\Gamma_J\left(\frac{\nu_{\text{inf}}}{2}\right)|\mathbf{\Psi'_{inf}}|^{\frac{\nu'_{\text{inf}}}{2}}} + w_{\text{vag}}\left(\frac{\lambda'_{\text{vag}}}{\lambda_{\text{vag}}}\right)^{-\frac{J}{2}} \frac{\Gamma_J\left(\frac{\nu'_{\text{vag}}}{2}\right)|\mathbf{\Psi_{vag}}|^{\frac{\nu_{\text{vag}}}{2}}}{\Gamma_J\left(\frac{\nu_{\text{vag}}}{2}\right)|\mathbf{\Psi'_{vag}}|^{\frac{\nu'_{\text{vag}}}{2}}}}$$

A.2.3 Posterior distribution for an MVN prior and MVN likelihood function

The following lemma is needed for Theorem A.6.

Lemma A.5 *Let* $\mathbf{S}_1$ *and* $\mathbf{S}_2$ *be symmetric, positive-definite matrices, and let* $\mathbf{v}_1$ *and* $\mathbf{v}_2$ *be vectors, then*

$$(\mathbf{S}_1^{-1}\mathbf{v}_1 + \mathbf{S}_2^{-1}\mathbf{v}_2)^\mathsf{T}(\mathbf{S}_1^{-1} + \mathbf{S}_2^{-1})^{-1}(\mathbf{S}_1^{-1}\mathbf{v}_1 + \mathbf{S}_2^{-1}\mathbf{v}_2) + (\mathbf{v}_1 - \mathbf{v}_2)^\mathsf{T}(\mathbf{S}_1 + \mathbf{S}_2)^{-1}(\mathbf{v}_1 - \mathbf{v}_2) = \mathbf{v}_1^\mathsf{T}\mathbf{S}_1^{-1}\mathbf{v}_1 + \mathbf{v}_2^\mathsf{T}\mathbf{S}_2^{-1}\mathbf{v}_2$$

Proof. Distribute all terms, apply the matrix inversion lemma (in two different forms), distribute again, cancelling terms and regroup.

$$\begin{aligned}
&(\mathbf{S}_1^{-1}\mathbf{v}_1 + \mathbf{S}_2^{-1}\mathbf{v}_2)^\mathsf{T}(\mathbf{S}_1^{-1} + \mathbf{S}_2^{-1})^{-1}(\mathbf{S}_1^{-1}\mathbf{v}_1 + \mathbf{S}_2^{-1}\mathbf{v}_2)\\
&\quad + (\mathbf{v}_1 - \mathbf{v}_2)^\mathsf{T}(\mathbf{S}_1 + \mathbf{S}_2)^{-1}(\mathbf{v}_1 - \mathbf{v}_2)\\
=&(\mathbf{S}_1^{-1}\mathbf{v}_1)^\mathsf{T}(\mathbf{S}_1^{-1} + \mathbf{S}_2^{-1})^{-1}(\mathbf{S}_1^{-1}\mathbf{v}_1)\\
&\quad + (\mathbf{S}_1^{-1}\mathbf{v}_1)^\mathsf{T}(\mathbf{S}_1^{-1} + \mathbf{S}_2^{-1})^{-1}(\mathbf{S}_2^{-1}\mathbf{v}_2)
\end{aligned}$$

© 2010 WILEY-VCH Verlag GmbH & Co. KGaA, Weinheim

$$
\begin{aligned}
&+ (\mathbf{S}_2^{-1}\mathbf{v}_2)^{\mathsf{T}}(\mathbf{S}_1^{-1} + \mathbf{S}_2^{-1})^{-1}(\mathbf{S}_1^{-1}\mathbf{v}_1) \\
&+ (\mathbf{S}_2^{-1}\mathbf{v}_2)^{\mathsf{T}}(\mathbf{S}_1^{-1} + \mathbf{S}_2^{-1})^{-1}(\mathbf{S}_2^{-1}\mathbf{v}_2) \\
&+ \mathbf{v}_1^{\mathsf{T}}(\mathbf{S}_1 + \mathbf{S}_2)^{-1}\mathbf{v}_1 \\
&- \mathbf{v}_1^{\mathsf{T}}(\mathbf{S}_1 + \mathbf{S}_2)^{-1}\mathbf{v}_2 \\
&- \mathbf{v}_2^{\mathsf{T}}(\mathbf{S}_1 + \mathbf{S}_2)^{-1}\mathbf{v}_1 \\
&+ \mathbf{v}_2^{\mathsf{T}}(\mathbf{S}_1 + \mathbf{S}_2)^{-1}\mathbf{v}_2 \\
=&\mathbf{v}_1^{\mathsf{T}}\mathbf{S}_1^{-1}\mathbf{v}_1 \\
&+ \mathbf{v}_2^{\mathsf{T}}\mathbf{S}_2^{-1}\mathbf{v}_2 \\
&+ \mathbf{v}_1^{\mathsf{T}}\left(\mathbf{S}_1^{-1} - \mathbf{S}_1^{-1}\mathbf{S}_2(\mathbf{S}_1 + \mathbf{S}_2)^{-1} - (\mathbf{S}_1 + \mathbf{S}_2)^{-1}\right)\mathbf{v}_2 \\
&+ \mathbf{v}_2^{\mathsf{T}}\left(\mathbf{S}_2^{-1} - \mathbf{S}_2^{-1}\mathbf{S}_1(\mathbf{S}_1 + \mathbf{S}_2)^{-1} - (\mathbf{S}_1 + \mathbf{S}_2)^{-1}\right)\mathbf{v}_1
\end{aligned}
$$

The sandwiched matrices in the third and fourth terms are the zero matrix (see below), hence they disappear and we are left with only the first two terms as required.

$$
\begin{aligned}
& & \mathbf{S}_1\mathbf{S}_1^{-1} &= \mathbf{I} \\
&\iff & \mathbf{S}_1^{-1}(\mathbf{S}_1 + \mathbf{S}_2) &= \mathbf{I} + \mathbf{S}_1^{-1}\mathbf{S}_2 \\
&\iff & \mathbf{S}_1^{-1} &= (\mathbf{S}_1 + \mathbf{S}_2)^{-1} + \mathbf{S}_1^{-1}\mathbf{S}_2(\mathbf{S}_1 + \mathbf{S}_2)^{-1}
\end{aligned}
$$

Similarly

$$
\mathbf{S}_2^{-1} = (\mathbf{S}_1 + \mathbf{S}_2)^{-1} + \mathbf{S}_2^{-1}\mathbf{S}_1(\mathbf{S}_1 + \mathbf{S}_2)^{-1}
$$

□

Theorem A.6 *Let $\boldsymbol{\mu}$, a vector of $J+K$ mean parameters be partitioned into two vectors, $\boldsymbol{\alpha}$ of length J and $\boldsymbol{\beta}$ of length K. Define a multivariate normal prior distribution on $\boldsymbol{\mu}$ such that $p(\boldsymbol{\mu}) = \varphi(\boldsymbol{\mu}; \boldsymbol{\mu}_0, \boldsymbol{\Sigma}_0) = \varphi((\begin{smallmatrix}\boldsymbol{\alpha}\\ \boldsymbol{\beta}\end{smallmatrix}); (\begin{smallmatrix}\boldsymbol{\alpha}_0\\ \boldsymbol{\beta}_0\end{smallmatrix}), (\begin{smallmatrix}\mathbf{A}_0 & \mathbf{B}_0\\ \mathbf{C}_0 & \mathbf{D}_0\end{smallmatrix}))$. Let $\boldsymbol{x}_i$ for $i = 1, 2, ..., n$ be i.i.d. outcome vectors observed on the first J dimensions. Let the variance of the $\boldsymbol{x}_i$ be known and equal to $\mathbf{A}$, and denote the mean of the $\boldsymbol{x}_i$ by $\bar{\boldsymbol{x}}$. Then for a multivariate normal likelihood function, the posterior distribution $p(\boldsymbol{\mu}|\boldsymbol{x})$ on the mean vector is given by*

$$
p(\boldsymbol{\mu}|\boldsymbol{x}) = \varphi\left(\begin{pmatrix}\boldsymbol{\alpha}\\ \boldsymbol{\beta}\end{pmatrix}; \begin{pmatrix}\boldsymbol{\alpha}_0' \\ \boldsymbol{\beta}_0 + \mathbf{C}_0\mathbf{A}_0^{-1}(\boldsymbol{\alpha}_0' - \boldsymbol{\alpha}_0)\end{pmatrix}, \begin{pmatrix}\mathbf{A}_0' & \mathbf{A}_0'\left(\mathbf{C}_0\mathbf{A}_0^{-1}\right)^{\mathsf{T}} \\ \mathbf{C}_0\mathbf{A}_0^{-1}\mathbf{A}_0' & \mathbf{S}_0 + \mathbf{C}_0\mathbf{A}_0^{-1}\mathbf{A}_0'\left(\mathbf{C}_0\mathbf{A}_0^{-1}\right)^{\mathsf{T}}\end{pmatrix}\right) \tag{5}
$$

where $\boldsymbol{\alpha}_0' = (\mathbf{A}_0^{-1} + n\mathbf{A}^{-1})^{-1}(\mathbf{A}_0^{-1}\boldsymbol{\alpha}_0 + n\mathbf{A}^{-1}\bar{\boldsymbol{x}})$, $\mathbf{A}_0' = (\mathbf{A}_0^{-1} + n\mathbf{A}^{-1})^{-1}$ and $\mathbf{S}_0 = \mathbf{D}_0 - \mathbf{C}_0\mathbf{A}_0^{-1}\mathbf{B}_0$.

Proof. Starting from Bayes' theorem with a $J+K$ dimension prior, and likelihood functions in J dimensions, the posterior can be calculated as follows

$$
\begin{aligned}
\text{posterior} &= \frac{\text{likelihood} \times \text{prior}}{\text{marginal likelihood}} \\
p(\boldsymbol{\mu}|\boldsymbol{x}) &= \frac{p(\boldsymbol{x}|\boldsymbol{\mu})p(\boldsymbol{\mu})}{p(\boldsymbol{x})}
\end{aligned}
$$

The marginal likelihood term $p(\boldsymbol{x})$ is derived in Theorem A.1 so all the terms are known and the posterior can be written

$$p(\boldsymbol{\mu}|\boldsymbol{x}) = \frac{\left[\prod_{i=1}^{n} \varphi(\boldsymbol{x}_i; \boldsymbol{\alpha}, \mathbf{A})\right] \varphi(\boldsymbol{\mu}; \boldsymbol{\mu_0}, \boldsymbol{\Sigma_0})}{(2\pi)^{\frac{J}{2}} \left|\frac{1}{n}\mathbf{A}\right|^{\frac{1}{2}} \left[\prod_{i=1}^{n} \varphi(\boldsymbol{x}_i; \bar{\boldsymbol{x}}, \mathbf{A})\right] \varphi\left(\bar{\boldsymbol{x}}; \boldsymbol{\alpha_0}, \mathbf{A_0} + \frac{1}{n}\mathbf{A}\right)} \tag{6}$$

First collecting the 2π terms from (6) it can be seen that the posterior has the expected $J+K$ dimensions

$$\frac{(2\pi)^{-\frac{nJ}{2}} (2\pi)^{-\frac{J+K}{2}}}{(2\pi)^{\frac{J}{2}} (2\pi)^{-\frac{nJ}{2}} (2\pi)^{-\frac{J}{2}}} = (2\pi)^{-\frac{J+K}{2}}$$

Then collecting the determinant terms from (6)

$$\begin{aligned}
\frac{|\mathbf{A}|^{-\frac{n}{2}} |\boldsymbol{\Sigma_0}|^{-\frac{1}{2}}}{\left|\frac{1}{n}\mathbf{A}\right|^{\frac{1}{2}} |\mathbf{A}|^{-\frac{n}{2}} \left|\mathbf{A_0} + \frac{1}{n}\mathbf{A}\right|^{-\frac{1}{2}}} &= \left(\frac{|\boldsymbol{\Sigma_0}|}{\left|\frac{1}{n}\mathbf{A}\right|^{-1} \left|\mathbf{A_0} + \frac{1}{n}\mathbf{A}\right|}\right)^{-\frac{1}{2}} \\
&= \left(\frac{|\boldsymbol{\Sigma_0}|}{\left|n\mathbf{A}^{-1}\mathbf{A_0} + \mathbf{I}\right|}\right)^{-\frac{1}{2}} \\
&= \left(|\boldsymbol{\Sigma_0}| \left|\left(\mathbf{I} + n\mathbf{A_0}\mathbf{A}^{-1}\right)^{-1}\right|\right)^{-\frac{1}{2}} \\
&= \left(|\mathbf{A_0}| \left|\mathbf{D_0} - \mathbf{C_0}\mathbf{A_0}^{-1}\mathbf{B_0}\right| \left|\left(\mathbf{I} + n\mathbf{A_0}\mathbf{A}^{-1}\right)^{-1}\right|\right)^{-\frac{1}{2}} \\
&= \left|\left(\mathbf{D_0} - \mathbf{C_0}\mathbf{A_0}^{-1}\mathbf{B_0}\right)\left(\mathbf{A_0}^{-1} + n\mathbf{A}^{-1}\right)^{-1}\right|^{-\frac{1}{2}} \\
&= \left|\mathbf{S_0}\mathbf{A}'_{\mathbf{0}}\right|^{-\frac{1}{2}}
\end{aligned}$$

The determinant of the covariance matrix in (5) is given by

$$\begin{aligned}
&\begin{vmatrix} \mathbf{A}'_{\mathbf{0}} & \mathbf{A}'_{\mathbf{0}}\mathbf{A_0}^{-1}\mathbf{B_0} \\ \mathbf{C_0}\mathbf{A_0}^{-1}\mathbf{A}'_{\mathbf{0}} & \mathbf{S_0} + \mathbf{C_0}\mathbf{A_0}^{-1}\mathbf{A}'_{\mathbf{0}}\mathbf{A_0}^{-1}\mathbf{B_0} \end{vmatrix} \\
&= \left|\mathbf{A}'_{\mathbf{0}}\right| \left|\mathbf{S_0} + \mathbf{C_0}\mathbf{A_0}^{-1}\mathbf{A}'_{\mathbf{0}}\mathbf{A_0}^{-1}\mathbf{B_0} - \mathbf{C_0}\mathbf{A_0}^{-1}\mathbf{A}'_{\mathbf{0}}\mathbf{A}'^{-1}_{\mathbf{0}}\mathbf{A}'_{\mathbf{0}}\mathbf{A_0}^{-1}\mathbf{B_0}\right| \\
&= \left|\mathbf{S_0}\mathbf{A}'_{\mathbf{0}}\right|
\end{aligned}$$

The determinant will be raised to the negative half power in the normal density function, so the determinant terms in the (5) and (6) are identical.

Finally collect the quadratic form terms inside the exponential functions in (6). First noticing that $\sum_1^n (\boldsymbol{x}_i - \boldsymbol{\alpha})^{\mathsf{T}} \mathbf{A}^{-1} (\boldsymbol{x}_i - \boldsymbol{\alpha}) - \sum_1^n (\boldsymbol{x}_i - \bar{\boldsymbol{x}})^{\mathsf{T}} \mathbf{A}^{-1} (\boldsymbol{x}_i - \bar{\boldsymbol{x}}) = (\bar{\boldsymbol{x}} - \boldsymbol{\alpha})^{\mathsf{T}} (\frac{1}{n}\mathbf{A})^{-1} (\bar{\boldsymbol{x}} - \boldsymbol{\alpha})$, the summed terms can be simplified

$$\begin{aligned}
&\sum_1^n (\boldsymbol{x}_i - \boldsymbol{\alpha})^{\mathsf{T}} \mathbf{A}^{-1} (\boldsymbol{x}_i - \boldsymbol{\alpha}) + (\boldsymbol{\mu} - \boldsymbol{\mu_0})^{\mathsf{T}} \boldsymbol{\Sigma_0}^{-1} (\boldsymbol{\mu} - \boldsymbol{\mu_0}) \\
&\quad - \sum_1^n (\boldsymbol{x}_i - \bar{\boldsymbol{x}})^{\mathsf{T}} \mathbf{A}^{-1} (\boldsymbol{x}_i - \bar{\boldsymbol{x}}) - (\bar{\boldsymbol{x}} - \boldsymbol{\alpha_0})^{\mathsf{T}} \left(\mathbf{A_0} + \tfrac{1}{n}\mathbf{A}\right)^{-1} (\bar{\boldsymbol{x}} - \boldsymbol{\alpha_0}) \\
&= (\bar{\boldsymbol{x}} - \boldsymbol{\alpha})^{\mathsf{T}} \left(\tfrac{1}{n}\mathbf{A}\right)^{-1} (\bar{\boldsymbol{x}} - \boldsymbol{\alpha}) + (\boldsymbol{\mu} - \boldsymbol{\mu_0})^{\mathsf{T}} \boldsymbol{\Sigma_0}^{-1} (\boldsymbol{\mu} - \boldsymbol{\mu_0}) \\
&\quad - (\bar{\boldsymbol{x}} - \boldsymbol{\alpha_0})^{\mathsf{T}} \left(\mathbf{A_0} + \tfrac{1}{n}\mathbf{A}\right)^{-1} (\bar{\boldsymbol{x}} - \boldsymbol{\alpha_0})
\end{aligned}$$

© 2010 WILEY-VCH Verlag GmbH & Co. KGaA, Weinheim

Decomposing the higher dimension term into its blocks.

$$
\begin{aligned}
&(\bar{\boldsymbol{x}}-\boldsymbol{\alpha})^{\mathsf{T}}\left(\tfrac{1}{n}\mathbf{A}\right)^{-1}(\bar{\boldsymbol{x}}-\boldsymbol{\alpha})\\
&\quad+(\boldsymbol{\alpha}-\boldsymbol{\alpha_0})^{\mathsf{T}}(\mathbf{A_0}^{-1}+\mathbf{A_0}^{-1}\mathbf{B_0}\mathbf{S_0}^{-1}\mathbf{C_0}\mathbf{A_0}^{-1})(\boldsymbol{\alpha}-\boldsymbol{\alpha_0})\\
&\quad-(\boldsymbol{\alpha}-\boldsymbol{\alpha_0})^{\mathsf{T}}\mathbf{A_0}^{-1}\mathbf{B_0}\mathbf{S_0}^{-1}(\boldsymbol{\beta}-\boldsymbol{\beta_0})\\
&\quad-(\boldsymbol{\beta}-\boldsymbol{\beta_0})^{\mathsf{T}}\mathbf{S_0}^{-1}\mathbf{C_0}\mathbf{A_0}^{-1}(\boldsymbol{\alpha}-\boldsymbol{\alpha_0})\\
&\quad+(\boldsymbol{\beta}-\boldsymbol{\beta_0})^{\mathsf{T}}\mathbf{S_0}^{-1}(\boldsymbol{\beta}-\boldsymbol{\beta_0})\\
&\quad-(\bar{\boldsymbol{x}}-\boldsymbol{\alpha_0})^{\mathsf{T}}\left(\mathbf{A_0}+\tfrac{1}{n}\mathbf{A}\right)^{-1}(\bar{\boldsymbol{x}}-\boldsymbol{\alpha_0})
\end{aligned}
$$

Finally expanding terms.

$$
\begin{aligned}
&(\bar{\boldsymbol{x}}-\boldsymbol{\alpha})^{\mathsf{T}}\left(\tfrac{1}{n}\mathbf{A}\right)^{-1}(\bar{\boldsymbol{x}}-\boldsymbol{\alpha})\\
&\quad+\boldsymbol{\alpha}^{\mathsf{T}}(\mathbf{A_0}^{-1}+\mathbf{A_0}^{-1}\mathbf{B_0}\mathbf{S_0}^{-1}\mathbf{C_0}\mathbf{A_0}^{-1})\boldsymbol{\alpha}\\
&\quad-\boldsymbol{\alpha}^{\mathsf{T}}(\mathbf{A_0}^{-1}+\mathbf{A_0}^{-1}\mathbf{B_0}\mathbf{S_0}^{-1}\mathbf{C_0}\mathbf{A_0}^{-1})\boldsymbol{\alpha_0}\\
&\quad-\boldsymbol{\alpha_0}^{\mathsf{T}}(\mathbf{A_0}^{-1}+\mathbf{A_0}^{-1}\mathbf{B_0}\mathbf{S_0}^{-1}\mathbf{C_0}\mathbf{A_0}^{-1})\boldsymbol{\alpha}\\
&\quad+\boldsymbol{\alpha_0}^{\mathsf{T}}(\mathbf{A_0}^{-1}+\mathbf{A_0}^{-1}\mathbf{B_0}\mathbf{S_0}^{-1}\mathbf{C_0}\mathbf{A_0}^{-1})\boldsymbol{\alpha_0}\\
&\quad-\boldsymbol{\alpha}^{\mathsf{T}}\mathbf{A_0}^{-1}\mathbf{B_0}\mathbf{S_0}^{-1}(\boldsymbol{\beta}-\boldsymbol{\beta_0})\\
&\quad+\boldsymbol{\alpha_0}^{\mathsf{T}}\mathbf{A_0}^{-1}\mathbf{B_0}\mathbf{S_0}^{-1}(\boldsymbol{\beta}-\boldsymbol{\beta_0})\\
&\quad-(\boldsymbol{\beta}-\boldsymbol{\beta_0})^{\mathsf{T}}\mathbf{S_0}^{-1}\mathbf{C_0}\mathbf{A_0}^{-1}\boldsymbol{\alpha}\\
&\quad+(\boldsymbol{\beta}-\boldsymbol{\beta_0})^{\mathsf{T}}\mathbf{S_0}^{-1}\mathbf{C_0}\mathbf{A_0}^{-1}\boldsymbol{\alpha_0}\\
&\quad+(\boldsymbol{\beta}-\boldsymbol{\beta_0})^{\mathsf{T}}\mathbf{S_0}^{-1}(\boldsymbol{\beta}-\boldsymbol{\beta_0})\\
&\quad+(\bar{\boldsymbol{x}}-\boldsymbol{\alpha_0})^{\mathsf{T}}\left(\mathbf{A_0}+\tfrac{1}{n}\mathbf{A}\right)^{-1}(\bar{\boldsymbol{x}}-\boldsymbol{\alpha_0})
\end{aligned}
$$

Compare this with the Gaussian kernel from (5),

$$
\begin{aligned}
&\begin{pmatrix}\boldsymbol{\alpha}-\boldsymbol{\alpha}'_{\mathbf{0}}\\ \boldsymbol{\beta}-\boldsymbol{\beta_0}-\mathbf{C_0}\mathbf{A_0}^{-1}(\boldsymbol{\alpha}'_{\mathbf{0}}-\boldsymbol{\alpha_0})\end{pmatrix}^{\mathsf{T}}\\
&\begin{pmatrix}\mathbf{A}'_{\mathbf{0}} & \mathbf{A}'_{\mathbf{0}}\mathbf{A_0}^{-1}\mathbf{B_0}\\ \mathbf{C_0}\mathbf{A_0}^{-1}\mathbf{A}'_{\mathbf{0}} & \mathbf{S_0}+\mathbf{C_0}\mathbf{A_0}^{-1}\mathbf{A}'_{\mathbf{0}}\mathbf{A_0}^{-1}\mathbf{B_0}\end{pmatrix}^{-1}\\
&\begin{pmatrix}\boldsymbol{\alpha}-\boldsymbol{\alpha}'_{\mathbf{0}}\\ \boldsymbol{\beta}-\boldsymbol{\beta_0}-\mathbf{C_0}\mathbf{A_0}^{-1}(\boldsymbol{\alpha}'_{\mathbf{0}}-\boldsymbol{\alpha_0})\end{pmatrix}\\
=&\begin{pmatrix}\boldsymbol{\alpha}-(\mathbf{A_0}^{-1}+n\mathbf{A}^{-1})^{-1}(\mathbf{A_0}^{-1}\boldsymbol{\alpha_0}+n\mathbf{A}^{-1}\bar{\boldsymbol{x}})\\ \boldsymbol{\beta}-\boldsymbol{\beta_0}-\mathbf{C_0}\mathbf{A_0}^{-1}[(\mathbf{A_0}^{-1}+n\mathbf{A}^{-1})^{-1}(\mathbf{A_0}^{-1}\boldsymbol{\alpha_0}+n\mathbf{A}^{-1}\bar{\boldsymbol{x}})-\boldsymbol{\alpha_0}]\end{pmatrix}^{\mathsf{T}}\\
&\begin{pmatrix}\mathbf{A_0}^{-1}+\mathbf{A_0}^{-1}\mathbf{B_0}\mathbf{S_0}^{-1}\mathbf{C_0}\mathbf{A_0}^{-1}+n\mathbf{A}^{-1} & -\mathbf{A_0}^{-1}\mathbf{B_0}\mathbf{S_0}^{-1}\\ -\mathbf{S_0}^{-1}\mathbf{C_0}\mathbf{A_0}^{-1} & \mathbf{S_0}^{-1}\end{pmatrix}\\
&\begin{pmatrix}\boldsymbol{\alpha}-(\mathbf{A_0}^{-1}+n\mathbf{A}^{-1})^{-1}(\mathbf{A_0}^{-1}\boldsymbol{\alpha_0}+n\mathbf{A}^{-1}\bar{\boldsymbol{x}})\\ \boldsymbol{\beta}-\boldsymbol{\beta_0}-\mathbf{C_0}\mathbf{A_0}^{-1}[(\mathbf{A_0}^{-1}+n\mathbf{A}^{-1})^{-1}(\mathbf{A_0}^{-1}\boldsymbol{\alpha_0}+n\mathbf{A}^{-1}\bar{\boldsymbol{x}})-\boldsymbol{\alpha_0}]\end{pmatrix}\\
=&\left(\boldsymbol{\alpha}-(\mathbf{A_0}^{-1}+n\mathbf{A}^{-1})^{-1}(\mathbf{A_0}^{-1}\boldsymbol{\alpha_0}+n\mathbf{A}^{-1}\bar{\boldsymbol{x}})\right)^{\mathsf{T}}\left(\mathbf{A_0}^{-1}+\mathbf{A_0}^{-1}\mathbf{B_0}\mathbf{S_0}^{-1}\mathbf{C_0}\mathbf{A_0}^{-1}+n\mathbf{A}^{-1}\right)\\
&\left(\boldsymbol{\alpha}-(\mathbf{A_0}^{-1}+n\mathbf{A}^{-1})^{-1}(\mathbf{A_0}^{-1}\boldsymbol{\alpha_0}+n\mathbf{A}^{-1}\bar{\boldsymbol{x}})\right)\\
&-\left(\boldsymbol{\alpha}-(\mathbf{A_0}^{-1}+n\mathbf{A}^{-1})^{-1}(\mathbf{A_0}^{-1}\boldsymbol{\alpha_0}+n\mathbf{A}^{-1}\bar{\boldsymbol{x}})\right)^{\mathsf{T}}\mathbf{A_0}^{-1}\mathbf{B_0}\mathbf{S_0}^{-1}
\end{aligned}
$$

© 2010 WILEY-VCH Verlag GmbH & Co. KGaA, Weinheim

$$\big(\boldsymbol{\beta}-\boldsymbol{\beta_0}-\mathbf{C_0}\mathbf{A_0}^{-1}[(\mathbf{A_0}^{-1}+n\mathbf{A}^{-1})^{-1}(\mathbf{A_0}^{-1}\boldsymbol{\alpha_0}+n\mathbf{A}^{-1}\bar{\boldsymbol{x}})-\boldsymbol{\alpha_0}]\big)$$
$$-\big(\boldsymbol{\beta}-\boldsymbol{\beta_0}-\mathbf{C_0}\mathbf{A_0}^{-1}[(\mathbf{A_0}^{-1}+n\mathbf{A}^{-1})^{-1}(\mathbf{A_0}^{-1}\boldsymbol{\alpha_0}+n\mathbf{A}^{-1}\bar{\boldsymbol{x}})-\boldsymbol{\alpha_0}]\big)^{\mathsf{T}}\mathbf{S_0}^{-1}\mathbf{C_0}\mathbf{A_0}^{-1}$$
$$\big(\boldsymbol{\alpha}-(\mathbf{A_0}^{-1}+n\mathbf{A}^{-1})^{-1}(\mathbf{A_0}^{-1}\boldsymbol{\alpha_0}+n\mathbf{A}^{-1}\bar{\boldsymbol{x}})\big)$$
$$+\big(\boldsymbol{\beta}-\boldsymbol{\beta_0}-\mathbf{C_0}\mathbf{A_0}^{-1}[(\mathbf{A_0}^{-1}+n\mathbf{A}^{-1})^{-1}(\mathbf{A_0}^{-1}\boldsymbol{\alpha_0}+n\mathbf{A}^{-1}\bar{\boldsymbol{x}})-\boldsymbol{\alpha_0}]\big)^{\mathsf{T}}\mathbf{S_0}^{-1}$$
$$\big(\boldsymbol{\beta}-\boldsymbol{\beta_0}-\mathbf{C_0}\mathbf{A_0}^{-1}[(\mathbf{A_0}^{-1}+n\mathbf{A}^{-1})^{-1}(\mathbf{A_0}^{-1}\boldsymbol{\alpha_0}+n\mathbf{A}^{-1}\bar{\boldsymbol{x}})-\boldsymbol{\alpha_0}]\big)$$

Expanding and cancelling in the quadratic forms the terms can be matched,

$$\boldsymbol{\alpha}^{\mathsf{T}}(\mathbf{A_0}^{-1}+\mathbf{A_0}^{-1}\mathbf{B_0}\mathbf{S_0}^{-1}\mathbf{C_0}\mathbf{A_0}^{-1})\boldsymbol{\alpha}+\boldsymbol{\alpha}^{\mathsf{T}}n\mathbf{A}^{-1}\boldsymbol{\alpha}$$
$$-\boldsymbol{\alpha}^{\mathsf{T}}(\mathbf{A_0}^{-1}\boldsymbol{\alpha_0}+n\mathbf{A}^{-1}\bar{\boldsymbol{x}})$$
$$-(\mathbf{A_0}^{-1}\boldsymbol{\alpha_0}+n\mathbf{A}^{-1}\bar{\boldsymbol{x}})^{\mathsf{T}}\boldsymbol{\alpha}$$
$$+(\mathbf{A_0}^{-1}\boldsymbol{\alpha_0}+n\mathbf{A}^{-1}\bar{\boldsymbol{x}})^{\mathsf{T}}(\mathbf{A_0}^{-1}+n\mathbf{A}^{-1})^{-1}(\mathbf{A_0}^{-1}\boldsymbol{\alpha_0}+n\mathbf{A}^{-1}\bar{\boldsymbol{x}})$$
$$-\boldsymbol{\alpha}^{\mathsf{T}}\mathbf{A_0}^{-1}\mathbf{B_0}\mathbf{S_0}^{-1}(\boldsymbol{\beta}-\boldsymbol{\beta_0})$$
$$-\boldsymbol{\alpha}^{\mathsf{T}}\mathbf{A_0}^{-1}\mathbf{B_0}\mathbf{S_0}^{-1}\mathbf{C_0}\mathbf{A_0}^{-1}\boldsymbol{\alpha_0}$$
$$-\boldsymbol{\alpha_0}^{\mathsf{T}}\mathbf{A_0}^{-1}\mathbf{B_0}\mathbf{S_0}^{-1}\mathbf{C_0}\mathbf{A_0}^{-1}\boldsymbol{\alpha}$$
$$+(\boldsymbol{\beta}-\boldsymbol{\beta_0})^{\mathsf{T}}\mathbf{S_0}^{-1}(\boldsymbol{\beta}-\boldsymbol{\beta_0})$$
$$+(\boldsymbol{\beta}-\boldsymbol{\beta_0})^{\mathsf{T}}\mathbf{S_0}^{-1}\mathbf{C_0}\mathbf{A_0}^{-1}\boldsymbol{\alpha_0}$$
$$+\boldsymbol{\alpha_0}^{\mathsf{T}}\mathbf{A_0}^{-1}\mathbf{B_0}\mathbf{S_0}^{-1}(\boldsymbol{\beta}-\boldsymbol{\beta_0})$$
$$+\boldsymbol{\alpha_0}^{\mathsf{T}}\mathbf{A_0}^{-1}\mathbf{B_0}\mathbf{S_0}^{-1}\mathbf{C_0}\mathbf{A_0}^{-1}\boldsymbol{\alpha_0}$$
$$-\bar{\boldsymbol{x}}^{\mathsf{T}}\big(\tfrac{1}{n}\mathbf{A}\big)^{-1}\bar{\boldsymbol{x}}$$

The final unmatched terms from (5) are

$$(\mathbf{A_0}^{-1}\boldsymbol{\alpha_0}+n\mathbf{A}^{-1}\bar{\boldsymbol{x}})^{\mathsf{T}}(\mathbf{A_0}^{-1}+n\mathbf{A}^{-1})^{-1}(\mathbf{A_0}^{-1}\boldsymbol{\alpha_0}+n\mathbf{A}^{-1}\bar{\boldsymbol{x}})$$
$$-\bar{\boldsymbol{x}}^{\mathsf{T}}\big(\tfrac{1}{n}\mathbf{A}\big)^{-1}\bar{\boldsymbol{x}}$$

Using Lemma A.5 these terms are equal to the final two terms from (6)

$$\boldsymbol{\alpha_0}^{\mathsf{T}}\mathbf{A_0}^{-1}\boldsymbol{\alpha_0}$$
$$-(\bar{\boldsymbol{x}}-\boldsymbol{\alpha_0})^{\mathsf{T}}\big(\mathbf{A_0}+\tfrac{1}{n}\mathbf{A}\big)^{-1}(\bar{\boldsymbol{x}}-\boldsymbol{\alpha_0})$$

The distributions in (5) and (6) are identical and so (5) is the posterior distribution. □

A.2.4 Posterior distribution for an NIW prior and MVN likelihood function

Theorem A.7 *Let $\boldsymbol{\mu}$, a vector of $J+K$ mean parameters be partitioned into two vectors, $\boldsymbol{\alpha}$ of length J and $\boldsymbol{\beta}$ of length K. Let $\boldsymbol{\Sigma}$ the matrix of covariance parameters be conformably partitioned into blocks, $\boldsymbol{\Sigma}=\left(\begin{smallmatrix}\mathbf{A} & \mathbf{B}\\ \mathbf{C} & \mathbf{D}\end{smallmatrix}\right)$. Define a normal-inverse-Wishart prior distribution on $(\boldsymbol{\mu},\boldsymbol{\Sigma})$ such that $p(\boldsymbol{\mu},\boldsymbol{\Sigma}) = \varphi(\boldsymbol{\mu};\boldsymbol{\mu_0},\boldsymbol{\Sigma}/\lambda_0)\,\omega(\boldsymbol{\Sigma};\boldsymbol{\Psi_0},\nu_0) = \varphi(\left(\begin{smallmatrix}\boldsymbol{\alpha}\\ \boldsymbol{\beta}\end{smallmatrix}\right);\left(\begin{smallmatrix}\boldsymbol{\alpha_0}\\ \boldsymbol{\beta_0}\end{smallmatrix}\right),\left(\begin{smallmatrix}\mathbf{A} & \mathbf{B}\\ \mathbf{C} & \mathbf{D}\end{smallmatrix}\right)/\lambda_0)\,\omega(\left(\begin{smallmatrix}\mathbf{A} & \mathbf{B}\\ \mathbf{C} & \mathbf{D}\end{smallmatrix}\right);\left(\begin{smallmatrix}\mathbf{A_0} & \mathbf{B_0}\\ \mathbf{C_0} & \mathbf{D_0}\end{smallmatrix}\right),\nu_0)$. Let $\boldsymbol{x}_i$ for $i = 1,2,...,n$ be i.i.d. outcome vectors observed on the first J dimensions. Denote the mean of the $\boldsymbol{x}_i$ by $\bar{\boldsymbol{x}}$. Then for a multivariate normal likelihood function, the posterior distribution $p(\boldsymbol{\mu},\boldsymbol{\Sigma}|\boldsymbol{x})$ on the mean*

© 2010 WILEY-VCH Verlag GmbH & Co. KGaA, Weinheim

vector and covariance matrix is given by

$$p(\boldsymbol{\mu}, \boldsymbol{\Sigma}|\boldsymbol{x}) = \varphi\left(\boldsymbol{\alpha}; \boldsymbol{\alpha}'_{\mathbf{0}}, \frac{\mathbf{A}}{\lambda'_0}\right) \omega\big(\mathbf{A}; \mathbf{A}'_{\mathbf{0}}, \nu'_0\big)\, \varphi\left(\boldsymbol{\beta}; \boldsymbol{\beta}_{\mathbf{0}} + \mathbf{C}\mathbf{A}^{-1}(\boldsymbol{\alpha} - \boldsymbol{\alpha}_{\mathbf{0}}), \frac{\mathbf{S}}{\lambda_0}\right) \omega(\mathbf{S}; \mathbf{S}_{\mathbf{0}}, \nu_0)$$
$$\frac{\Gamma_J\left(\frac{\nu'_0}{2}\right)\Gamma_K\left(\frac{\nu_0}{2}\right)\Gamma_J\left(\frac{\nu_0-K}{2}\right)|\mathbf{A}_{\mathbf{0}}|^{\frac{K}{2}}}{\Gamma_{J+K}\left(\frac{\nu_0}{2}\right)\Gamma_J\left(\frac{\nu'_0-K}{2}\right)\left|\mathbf{A}'_{\mathbf{0}}\right|^{\frac{K}{2}}} |\mathbf{A}|^{-\frac{K}{2}} |\mathbf{S}|^{-\frac{J}{2}} \tag{7}$$
$$\exp\left[-\frac{1}{2}\operatorname{tr}\Big(\big(\mathbf{C}\mathbf{A}^{-1} - \mathbf{C}_{\mathbf{0}}\mathbf{A}_{\mathbf{0}}^{-1}\big)\mathbf{A}_{\mathbf{0}}\big(\mathbf{C}\mathbf{A}^{-1} - \mathbf{C}_{\mathbf{0}}\mathbf{A}_{\mathbf{0}}^{-1}\big)^{\mathsf{T}}\mathbf{S}^{-1}\Big)\right]$$

where $\boldsymbol{\alpha}'_{\mathbf{0}} = \dfrac{\lambda_0\boldsymbol{\alpha}_{\mathbf{0}} + n\bar{\boldsymbol{x}}}{\lambda_0 + n}$, $\mathbf{A}'_{\mathbf{0}} = \mathbf{A}_{\mathbf{0}} + \left[\sum_1^n(\boldsymbol{x}_i - \bar{\boldsymbol{x}})(\boldsymbol{x}_i - \bar{\boldsymbol{x}})^{\mathsf{T}}\right] + \dfrac{\lambda_0 n}{\lambda_0 + n}(\boldsymbol{\alpha}_{\mathbf{0}} - \bar{\boldsymbol{x}})(\boldsymbol{\alpha}_{\mathbf{0}} - \bar{\boldsymbol{x}})^{\mathsf{T}}$, $\lambda'_0 = \lambda_0 + n$, $\nu'_0 = \nu_0 + n$, $\mathbf{S} = \mathbf{D} - \mathbf{C}\mathbf{A}^{-1}\mathbf{B}$ *and* $\mathbf{S}_{\mathbf{0}} = \mathbf{D}_{\mathbf{0}} - \mathbf{C}_{\mathbf{0}}\mathbf{A}_{\mathbf{0}}^{-1}\mathbf{B}_{\mathbf{0}}$.

Proof.

$$\text{posterior} = \frac{\text{likelihood} \times \text{prior}}{\text{marginal likelihood}}$$
$$p(\boldsymbol{\mu}, \boldsymbol{\Sigma}|\boldsymbol{x}) = \frac{p(\boldsymbol{x}|\boldsymbol{\mu}, \boldsymbol{\Sigma})p(\boldsymbol{\mu}, \boldsymbol{\Sigma})}{p(\boldsymbol{x})}$$

The marginal likelihood term $p(\boldsymbol{x})$ is derived in Theorem A.3, so all the terms are known and the posterior can be written

$$p(\boldsymbol{\mu}, \boldsymbol{\Sigma}|\boldsymbol{x}) = \frac{\left[\prod_{i=1}^{n}\varphi(\boldsymbol{x}_i; \boldsymbol{\alpha}, \mathbf{A})\right] \varphi\left(\boldsymbol{\mu}; \boldsymbol{\mu}_{\mathbf{0}}, \frac{\boldsymbol{\Sigma}}{\lambda_0}\right) \omega(\boldsymbol{\Sigma}, \boldsymbol{\Psi}_{\mathbf{0}}, \nu_0)}{\pi^{-\frac{nJ}{2}}\left(\frac{\lambda'_0}{\lambda_0}\right)^{-\frac{J}{2}} \dfrac{\Gamma_J\left(\frac{\nu'_0-K}{2}\right)|\mathbf{A}_{\mathbf{0}}|^{\frac{\nu_0-K}{2}}}{\Gamma_J\left(\frac{\nu_0-K}{2}\right)\left|\mathbf{A}'_{\mathbf{0}}\right|^{\frac{\nu'_0-K}{2}}}} \tag{8}$$

Collecting terms from (8), the normalising constant for the posterior can be written

$$(2\pi)^{-\frac{nJ}{2}} \frac{\lambda_0^{\frac{J+K}{2}}|\boldsymbol{\Psi}_{\mathbf{0}}|^{\frac{\nu_0}{2}}}{(2\pi)^{\frac{J+K}{2}}2^{\frac{\nu_0(J+K)}{2}}\Gamma_{J+K}\left(\frac{\nu_0}{2}\right)} \cdot \frac{\Gamma_J\left(\frac{\nu_0-K}{2}\right)\left|\mathbf{A}'_{\mathbf{0}}\right|^{\frac{\nu'_0-K}{2}}}{\pi^{-\frac{nJ}{2}}\left(\frac{\lambda'_0}{\lambda_0}\right)^{-\frac{J}{2}}\Gamma_J\left(\frac{\nu'_0-K}{2}\right)|\mathbf{A}_{\mathbf{0}}|^{\frac{\nu_0-K}{2}}}$$
$$= \frac{{\lambda'_0}^{\frac{J}{2}}\left|\mathbf{A}'_{\mathbf{0}}\right|^{\frac{\nu'_0}{2}}}{(2\pi)^{\frac{J}{2}}2^{\frac{\nu'_0 J}{2}}\Gamma_J\left(\frac{\nu'_0}{2}\right)} \cdot \frac{\lambda_0^{\frac{K}{2}}|\mathbf{S}_{\mathbf{0}}|^{\frac{\nu_0}{2}}}{(2\pi)^{\frac{K}{2}}2^{\frac{\nu_0 K}{2}}\Gamma_K\left(\frac{\nu_0}{2}\right)} \cdot \frac{\Gamma_J\left(\frac{\nu'_0}{2}\right)\Gamma_K\left(\frac{\nu_0}{2}\right)\Gamma_J\left(\frac{\nu_0-K}{2}\right)|\mathbf{A}_{\mathbf{0}}|^{\frac{K}{2}}}{\Gamma_{J+K}\left(\frac{\nu_0}{2}\right)\Gamma_J\left(\frac{\nu'_0-K}{2}\right)\left|\mathbf{A}'_{\mathbf{0}}\right|^{\frac{K}{2}}}$$

Then collecting the variable terms from (8), the non-normalised density function can be written

$$|\mathbf{A}|^{-\frac{n}{2}}\exp\left[-\frac{1}{2}\sum_{i=1}^{n}(\boldsymbol{x}_i - \boldsymbol{\alpha})^{\mathsf{T}}\mathbf{A}^{-1}(\boldsymbol{x}_i - \boldsymbol{\alpha})\right]$$
$$|\boldsymbol{\Sigma}|^{-\frac{\nu_0+J+K+2}{2}}\exp\left[-\frac{1}{2}\operatorname{tr}(\boldsymbol{\Psi}_{\mathbf{0}}\boldsymbol{\Sigma}^{-1}) - \frac{\lambda_0}{2}(\boldsymbol{\mu} - \boldsymbol{\mu}_{\mathbf{0}})^{\mathsf{T}}\boldsymbol{\Sigma}^{-1}(\boldsymbol{\mu} - \boldsymbol{\mu}_{\mathbf{0}})\right]$$
$$= |\mathbf{A}|^{-\frac{n}{2}}\exp\left[-\frac{1}{2}\sum_{i=1}^{n}(\boldsymbol{x}_i - \boldsymbol{\alpha})^{\mathsf{T}}\mathbf{A}^{-1}(\boldsymbol{x}_i - \boldsymbol{\alpha})\right]|\mathbf{A}|^{-\frac{\nu_0+J+K+2}{2}}|\mathbf{S}|^{-\frac{\nu_0+J+K+2}{2}}$$

© 2010 WILEY-VCH Verlag GmbH & Co. KGaA, Weinheim

$$
\begin{aligned}
&\exp\left[-\frac{1}{2}\operatorname{tr}(\mathbf{A_0}\mathbf{A}^{-1}+\mathbf{A_0}\mathbf{A}^{-1}\mathbf{B}\mathbf{S}^{-1}\mathbf{C}\mathbf{A}^{-1}-\mathbf{B_0}\mathbf{S}^{-1}\mathbf{C}\mathbf{A}^{-1}-\mathbf{C_0}\mathbf{A}^{-1}\mathbf{B}\mathbf{S}^{-1}+\mathbf{D_0}\mathbf{S}^{-1})\right]\\
&\exp\left[-\frac{\lambda_0}{2}(\boldsymbol{\alpha}-\boldsymbol{\alpha_0})^\mathsf{T}\mathbf{A}^{-1}(\boldsymbol{\alpha}-\boldsymbol{\alpha_0})-\frac{\lambda_0}{2}(\boldsymbol{\alpha}-\boldsymbol{\alpha_0})^\mathsf{T}\mathbf{A}^{-1}\mathbf{B}\mathbf{S}^{-1}\mathbf{C}\mathbf{A}^{-1}(\boldsymbol{\alpha}-\boldsymbol{\alpha_0})\right]\\
&\exp\left[\frac{\lambda_0}{2}(\boldsymbol{\alpha}-\boldsymbol{\alpha_0})^\mathsf{T}\mathbf{A}^{-1}\mathbf{B}\mathbf{S}^{-1}(\boldsymbol{\beta}-\boldsymbol{\beta_0})+\frac{\lambda_0}{2}(\boldsymbol{\beta}-\boldsymbol{\beta_0})^\mathsf{T}\mathbf{S}^{-1}\mathbf{C}\mathbf{A}^{-1}(\boldsymbol{\alpha}-\boldsymbol{\alpha_0})\right]\\
&\exp\left[-\frac{\lambda_0}{2}(\boldsymbol{\beta}-\boldsymbol{\beta_0})^\mathsf{T}\mathbf{S}^{-1}(\boldsymbol{\beta}-\boldsymbol{\beta_0})\right]\\
=&|\mathbf{A}|^{-\frac{\nu_0'+J+2}{2}}\exp\left[-\frac{1}{2}\operatorname{tr}(\mathbf{A_0'}\mathbf{A}^{-1})-\frac{\lambda_0'}{2}(\boldsymbol{\alpha}-\boldsymbol{\alpha_0'})^\mathsf{T}\mathbf{A}^{-1}(\boldsymbol{\alpha}-\boldsymbol{\alpha_0'})\right]\\
&|\mathbf{S}|^{-\frac{\nu_0+K+2}{2}}\exp\left[-\frac{1}{2}\operatorname{tr}\big(\mathbf{S_0}\mathbf{S}^{-1}\big)-\frac{\lambda_0}{2}\big(\boldsymbol{\beta}-\boldsymbol{\beta_0}-\mathbf{C}\mathbf{A}^{-1}(\boldsymbol{\alpha}-\boldsymbol{\alpha_0})\big)^\mathsf{T}\mathbf{S}^{-1}\big(\boldsymbol{\beta}-\boldsymbol{\beta_0}-\mathbf{C}\mathbf{A}^{-1}(\boldsymbol{\alpha}-\boldsymbol{\alpha_0})\big)\right]\\
&|\mathbf{A}|^{-\frac{K}{2}}|\mathbf{S}|^{-\frac{J}{2}}\exp\left[-\frac{1}{2}\operatorname{tr}\Big(\big(\mathbf{C}\mathbf{A}^{-1}-\mathbf{C_0}\mathbf{A_0}^{-1}\big)\mathbf{A_0}\big(\mathbf{C}\mathbf{A}^{-1}-\mathbf{C_0}\mathbf{A_0}^{-1}\big)^\mathsf{T}\mathbf{S}^{-1}\Big)\right]
\end{aligned}
$$

The posterior density function can therefore be written as (7)

$$
\begin{aligned}
p(\boldsymbol{\mu},\boldsymbol{\Sigma}|\boldsymbol{x}) =&\ \varphi\left(\boldsymbol{\alpha};\boldsymbol{\alpha_0'},\frac{\mathbf{A}}{\lambda_0'}\right)\omega\big(\mathbf{A};\mathbf{A_0'},\nu_0'\big)\,\varphi\left(\boldsymbol{\beta};\boldsymbol{\beta_0}+\mathbf{C}\mathbf{A}^{-1}(\boldsymbol{\alpha}-\boldsymbol{\alpha_0}),\frac{\mathbf{S}}{\lambda_0}\right)\omega(\mathbf{S};\mathbf{S_0},\nu_0)\\
&\frac{\Gamma_J\left(\frac{\nu_0'}{2}\right)\Gamma_K\left(\frac{\nu_0}{2}\right)\Gamma_J\left(\frac{\nu_0-K}{2}\right)|\mathbf{A_0}|^{\frac{K}{2}}}{\Gamma_{J+K}\left(\frac{\nu_0}{2}\right)\Gamma_J\left(\frac{\nu_0'-K}{2}\right)\big|\mathbf{A_0'}\big|^{\frac{K}{2}}}|\mathbf{A}|^{-\frac{K}{2}}|\mathbf{S}|^{-\frac{J}{2}}\\
&\exp\left[-\frac{1}{2}\operatorname{tr}\Big(\big(\mathbf{C}\mathbf{A}^{-1}-\mathbf{C_0}\mathbf{A_0}^{-1}\big)\mathbf{A_0}\big(\mathbf{C}\mathbf{A}^{-1}-\mathbf{C_0}\mathbf{A_0}^{-1}\big)^\mathsf{T}\mathbf{S}^{-1}\Big)\right]
\end{aligned}
$$

□

Remark A.8 When $K = 0$, *i.e.* all visits are observed, the density function is the usual updated NIW posterior distribution.

$$p(\boldsymbol{\mu},\boldsymbol{\Sigma}|\boldsymbol{x}) = \varphi\left(\boldsymbol{\alpha};\boldsymbol{\alpha_0'},\frac{\mathbf{A}}{\lambda_0'}\right)\omega\big(\mathbf{A};\mathbf{A_0'},\nu_0'\big)$$

When $J = 0$, *i.e.* there are no observed visits, the density function reduces to the NIW prior distribution.

$$p(\boldsymbol{\mu},\boldsymbol{\Sigma}|\boldsymbol{x}) = p(\boldsymbol{\mu},\boldsymbol{\Sigma}) = \varphi\left(\boldsymbol{\beta};\boldsymbol{\beta_0},\frac{\mathbf{D}}{\lambda_0}\right)\omega(\mathbf{D};\mathbf{D_0},\nu_0)$$

A.3 Additional simulation study results

© 2010 WILEY-VCH Verlag GmbH & Co. KGaA, Weinheim

Table 1 Simulation Study Results for Marginal Estimates at Week 104.

				Linear Prior Data and Intercept-Slope Analysis Model 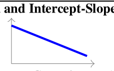					Linear Prior Data and By-Visit Analysis Model 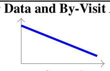					Dog-Leg Prior Data and By-Visit Analysis Model 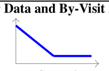				
	Prior Weight w_{inf}	True Active Mean (mL)	True Control Mean (mL)	Average Posterior Weight w'_{inf}	Average Control Bias (mL)	Average Control SE (mL)	Average ESS of Prior	Type 1 Error or Power	Average Posterior Weight w'_{inf}	Average Control Bias (mL)	Average Control SE (mL)	Average ESS of Prior	Type 1 Error or Power	Average Posterior Weight w'_{inf}	Average Control Bias (mL)	Average Control SE (mL)	Average ESS of Prior	Type 1 Error or Power
**Null Effect Current Data**																		
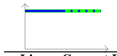	0.25	3000	3000	0.04	-7	73	-2	0.04	0.68	-137	123	62	0.21	0.00	-6	239	1	0.00
	0.75			0.13	-16	74	-5	0.05	0.85	-147	85	112	0.34	0.00	-5	239	1	0.00
**Linear Current Data**																		
			2950	0.21	-18	73	1	0.12	0.93	-114	66	137	0.46	0.00	20	238	1	0.00
	0.25	3000	2900	0.55	-33	65	43	0.32	0.99	-78	48	183	0.59	0.05	43	232	2	0.00
			2850	0.82	-26	54	112	0.55	>0.99	-39	45	196	0.66	0.29	55	196	14	0.04
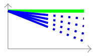			2800	0.90	$0^{\dagger}$	50	143	0.69	>0.99	$0^{\dagger}$	45	198	0.71	0.69	63	123	62	0.21
			2950	0.46	-42	70	23	0.20	0.98	-117	52	175	0.52	0.01	19	237	1	0.00
	0.75	3000	2900	0.81	-54	57	104	0.44	>0.99	-78	46	196	0.61	0.14	37	219	6	0.01
			2850	0.96	-34	48	170	0.63	>0.99	-39	45	199	0.67	0.50	41	159	38	0.11
			2800	0.99	$0^{\dagger}$	46	188	0.72	>0.99	$0^{\dagger}$	45	200	0.72	0.85	53	85	112	0.34
**Dog-Leg Current Data**																		
			2950	0.55	-83	65	44	0.32	0.99	-128	48	183	0.59	0.05	-7	232	2	0.00
	0.25	3000	2900	0.90	$-100^{\ddagger}$	50	142	0.69	>0.99	$-100^{\ddagger}$	45	198	0.72	0.69	-37	123	63	0.21
			2850	0.55	-117	65	43	0.85	0.99	-72	48	183	0.80	0.99	-28	48	183	0.60
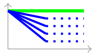			2800	0.04	-194	73	-2	0.98	0.69	-63	123	63	0.51	>0.99	$0^{\dagger}$	45	198	0.71
			2950	0.82	-104	57	104	0.45	>0.99	-129	46	341	0.61	0.14	-13	219	6	0.01
	0.75	3000	2900	0.99	$-100^{\ddagger}$	46	188	0.72	>0.99	$-100^{\ddagger}$	45	348	0.72	0.85	-47	85	112	0.34
			2850	0.82	-96	57	105	0.84	>0.99	-72	46	341	0.81	>0.99	-28	46	196	0.61
			2800	0.13	-184	74	-5	0.97	0.85	-53	85	194	0.70	>0.99	$0^{\dagger}$	45	200	0.72
**Double-Dog-Leg Current Data**																		
			2950	0.25	-26	72	4	0.14	0.92	-113	71	129	0.44	0.00	20	238	1	0.00
	0.25	3000	2900	0.64	-45	62	61	0.38	0.93	-76	66	137	0.51	0.03	44	235	2	0.00
			2850	0.86	-34	52	125	0.60	0.77	-36	103	82	0.38	0.07	68	230	3	0.00
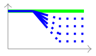			2800	0.86	-9	53	124	0.72	0.30	0	193	16	0.09	0.05	95	232	2	0.00
			2950	0.52	-52	69	32	0.23	0.97	-116	54	170	0.52	0.02	19	237	1	0.00
	0.75	3000	2900	0.87	-62	54	125	0.50	0.98	-78	52	175	0.58	0.09	40	226	4	0.01
			2850	0.97	-38	47	179	0.66	0.91	-38	71	133	0.53	0.17	62	214	8	0.02
			2800	0.97	-6	47	178	0.74	0.51	0	154	42	0.23	0.14	91	219	6	0.02

†no prior-current data conflict ‡no short-term prior-current data conflict followed by unobserved long-term conflict

References

Bernardo, J. M. and Smith, A. (2000). *Bayesian Theory*. John Wiley & Sons Ltd., Chichester.

Gupta, A. K. and Nagar, D. K. (2018). *Matrix variate distributions*. Chapman and Hall/CRC.

R Core Team (2025). *R: A Language and Environment for Statistical Computing*. R Foundation for Statistical Computing, Vienna, Austria. Version 4.5.0 (2025-04-11).

Stan Development Team (2025). RStan: the R interface to Stan. R package version 2.32.7.

Venables, W. N. and Ripley, B. D. (2002). *Modern Applied Statistics with S*. Springer, New York, fourth edition. ISBN 0-387-95457-0.

© 2010 WILEY-VCH Verlag GmbH & Co. KGaA, Weinheim
